# Selective Modulation of Interhemispheric Functional Connectivity by HD-tACS Shapes Perception

**DOI:** 10.1371/journal.pbio.1002031

**Published:** 2014-12-30

**Authors:** Randolph F. Helfrich, Hannah Knepper, Guido Nolte, Daniel Strüber, Stefan Rach, Christoph S. Herrmann, Till R. Schneider, Andreas K. Engel

**Affiliations:** 1 Department of Neurophysiology and Pathophysiology, University Medical Center Hamburg-Eppendorf, Hamburg, Germany; 2 Experimental Psychology Lab, Center for Excellence ‘Hearing4all’, European Medical School, University of Oldenburg, Oldenburg, Germany; 3 Research Center Neurosensory Science, University of Oldenburg, Oldenburg, Germany; 4 Department of Epidemiological Methods and Etiologic Research, Leibniz Institute for Prevention Research and Epidemiology – BIPS, Bremen, Germany; Radboud University Nijmegen, Netherlands

## Abstract

Oscillatory neuronal synchronization between cortical areas has been suggested to constitute a flexible mechanism to coordinate information flow in the human cerebral cortex. However, it remains unclear whether synchronized neuronal activity merely represents an epiphenomenon or whether it is causally involved in the selective gating of information. Here, we combined bilateral high-density transcranial alternating current stimulation (HD-tACS) at 40 Hz with simultaneous electroencephalographic (EEG) recordings to study immediate electrophysiological effects during the selective entrainment of oscillatory gamma-band signatures. We found that interhemispheric functional connectivity was modulated in a predictable, phase-specific way: In-phase stimulation enhanced synchronization, anti-phase stimulation impaired functional coupling. Perceptual correlates of these connectivity changes were found in an ambiguous motion task, which strongly support the functional relevance of long-range neuronal coupling. Additionally, our results revealed a decrease in oscillatory alpha power in response to the entrainment of gamma band signatures. This finding provides causal evidence for the antagonistic role of alpha and gamma oscillations in the parieto-occipital cortex and confirms that the observed gamma band modulations were physiological in nature. Our results demonstrate that synchronized cortical network activity across several spatiotemporal scales is essential for conscious perception and cognition.

## Abstract 2

This transcranial stimulation study shows that selective modulation of synchronized neuronal activity between the hemispheres of the brain can affect conscious perception.

## Author Summary

Brain activity is profoundly rhythmic and exhibits seemingly random fluctuations across a very broad frequency range (<0.1 Hz to >600 Hz). Recently, it has become evident that these brain rhythms are not just a generic sign of the brain-at-work, but actually reflect a highly flexible mechanism for information encoding and transfer. In particular, it has been suggested that oscillatory synchronization between different areas of the cortex underlies the establishment of task-relevant networks. Here, we investigated whether gamma-band synchronization (∼40 Hz) is causally involved in the integration between the two brain hemispheres of alternating visual tokens into a coherent motion percept. We utilized transcranial alternating current stimulation (tACS), a novel non-invasive brain stimulation technique, which allows frequency-specific entrainment of cortical areas. In a combined tACS-electroencephalography study, we selectively up- and down-regulated interhemispheric coherence, resulting in a directed bias in apparent motion perception: Increased interhemispheric connectivity sustained the horizontal motion percept, while decreased connectivity reinforced the vertical percept. Thus, our data suggest that the level of interhemispheric gamma-band coherence directly influenced the instantaneous motion percept. From these results, we conclude that synchronized neuronal activity is essential for conscious perception and cognition.

## Introduction

Synchronization of oscillatory brain activity on multiple temporal scales across distant cortical regions is thought to constitute a key mechanism for conscious perception and cognition in humans [1]–[3]. Recently, it has become evident that synchronized cortical networks are dynamically established during cognitive processing to selectively route information to task-relevant cortical sites [4],[5]. In particular, it has been shown that cortical information flow can selectively be controlled by shifting phase relations between cell groups oscillating at similar frequencies [6]. In the visual system, synchronized gamma-band activity, mediated over cortio-cortical callosal connections, might facilitate feature integration across both visual hemifields [7].

However, most evidence is still correlative in nature [3]. As a consequence, it remains unclear whether the observed rhythmic synchronization patterns merely represent concurrent neuronal activity or whether they are causally relevant for the information flow within cortical networks. The frequency-specific modulation of phase relations with subsequent behavioral alterations would constitute an unequivocal confirmation for the functional role of synchronization processes in large-scale neuronal networks. Up to now, it has been considered difficult to selectively manipulate rhythmic brain activity in the human cerebral cortex. Recently, novel methods for entrainment of perceptually relevant brain oscillations have become available [8]. In particular, transcranial alternating current stimulation (tACS) has been shown to entrain cortical oscillations in a frequency-specific manner [9]. Phase-dependent tACS effects have been demonstrated in a variety of human [10],[11], animal [12],[13], and modeling studies [14], making it a prime candidate to selectively modulate phase relationships in distant cortical regions [15]. Optimized stimulation electrode montages and novel multi-electrode setups now allow a selective cortical stimulation [16],[17].

**Figure 1:**
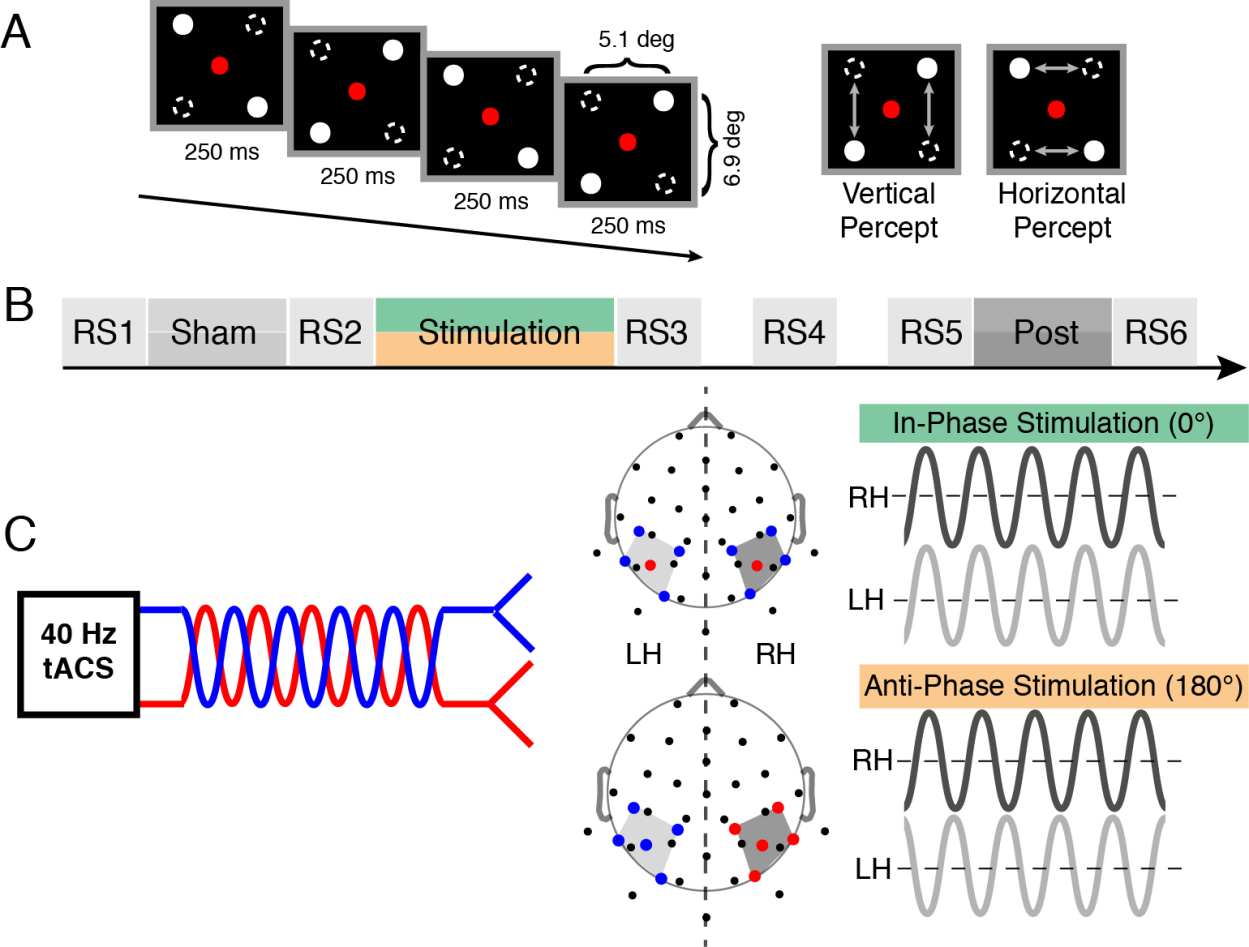
Procedure and tACS electrode montage. (A) Left: Alternating presentation of two displays with diagonal tokens. Right: All subjects perceived either vertical or horizontal motion with spontaneous perceptual reversals. (B) The experiment was conducted on two separate days (for in- and anti-phase session). The SAM was presented during sham, stimulation, and post blocks and was interleaved with six RS recordings. RS3/4 and RS4/5 were separated by 10-minute intervals. (C) Electrode montage: The output signals of the tACS-stimulator were split with several Y-connectors and fed into 10 Ag/AgCl electrodes (common impedance <5 kΩ), which were positioned on the cortex to create an in-phase (0° phase difference between hemispheres, green) and anti-phase (180° phase difference, orange) setup. Red and blue lines/dots depict the connection to the respective stimulator channels. Right and left hemispheres (RH/LH) are depicted in dark and light grey (see also Figure S1).

The goal of this study was to test whether perceptually relevant neuronal synchronization in large-scale neuronal networks can selectively be modulated with weak electric currents. Recent studies investigating tACS effects on long-range functional coupling remained equivocal and did not provide conclusive electrophysiological evidence for the causal role of synchronized oscillatory activity [15],[18]. Here, we focused on the role of interhemispheric gamma-band coherence over parieto-occipital areas during ambiguous motion perception. To this end, we utilized the well-known ambiguous stroboscopic alternative motion (SAM) paradigm (Figure 1A) [18]. In this paradigm, a physically identical stimulus can be perceived as either horizontally or vertically moving [19]. This perceptual bi-stability makes the stimulus ideally suited to assess percept related network differences. The perceived motion direction might alternate spontaneously depending on the level of interhemispheric gamma-band coherence over motion sensitive cortical areas [20],[21], and might be mediated via cortico-cortical callosal fibers [22].

To investigate the functional role of interhemispheric gamma-band coherence in ambiguous motion perception, we combined multi-focal 40 Hz high density (HD)-tACS at opposite polarities (in-/anti-phase stimulation) with concomitant electroencephalographic (EEG) recordings to investigate oscillatory brain activity during stimulation. We expected that the different stimulation protocols might bias network synchrony in opposite directions and therefore facilitate or impair interhemispheric integration of the visual tokens into the horizontal percept.

## Results

**Figure 2:**
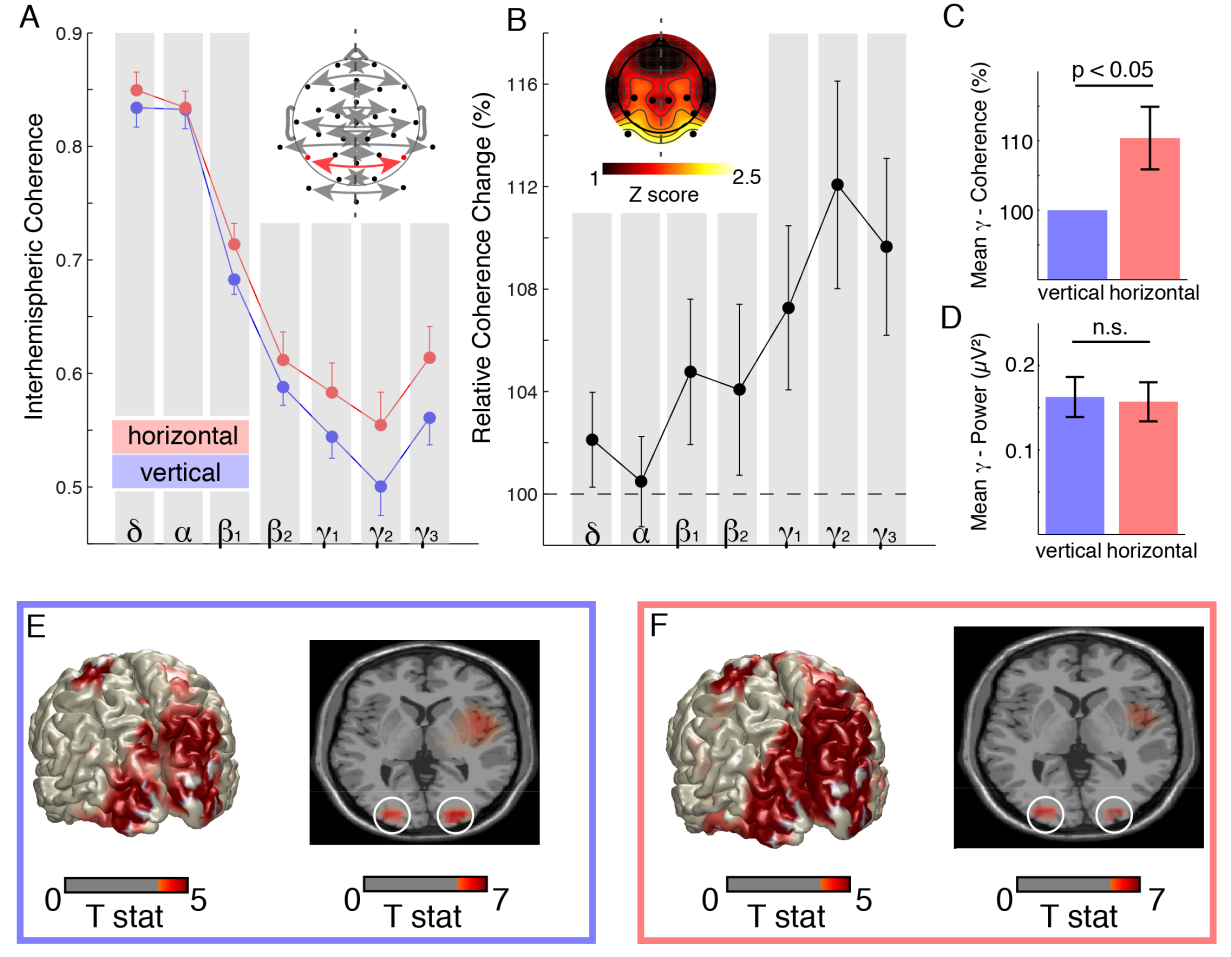
Stroboscopic alternative motion stimulus. (A) Grand-mean coherence average (sham and post, both sessions) over electrode pair-of-interest (inset: electrode layout, pair-of-interest highlighted in red, dashed lines in topographies indicate the symmetry axis) during vertical (blue) and horizontal (red) motion perception (mean ± SEM). See also Data S1. (B) Relative coherence increases during horizontal motion perception. The topography depicts the statistical map of the coherence increase (black dots highlight the significant electrode cluster). (C) Gamma-coherence (γ_1_–γ_3_) increase by (+9.5%±3.2%) during horizontal motion perception. (D) Gamma-band power did not differ significantly between both percepts at the same electrodes. (E) Source reconstruction of gamma activity (60±10 Hz) for the vertical percept as compared to the resting state baseline. (F) Source reconstruction for the horizontal percept as compared to baseline.

Participants (*n* = 14) were presented with the SAM stimulus (Figure 1A) on two separate days (sessions) in three different conditions (sham, stimulation, and post) (Figure 1B). In each session either in- or anti-phase tACS was applied. In case of horizontal motion perception, visual information from both visual hemifields has to be integrated and thus might be reflected in enhanced functional connectivity between both hemispheres [20]. We directly assessed coherence values for 13 interhemispheric electrode pairs (Figure 2A inset, electrode pair of interest in red) and seven *frequency*-bands of interest: delta/theta (1–7 Hz), alpha (8–12 Hz), beta_1_ (13–25 Hz), beta_2_ (26–35 Hz), gamma_1_ (36–45 Hz), gamma_2_ (46–70 Hz), and gamma_3_ (71–100 Hz).

Consistent with results from a previous study [20], we found a significant increase in gamma-band (γ_1–3_) coherence over extrastriate visual areas during horizontal motion perception (+9.5%±3.2%; t_13_ = −2.9, *p*<0.05) (Figure 2A–2C). Importantly, this effect was confined to parieto-occipital areas (cluster test: *p* = 0.001) (Figure 2B, inset) and not accompanied by any differences in gamma power between both percepts (Figure 2D; t_13_ = 1.3, *p* = 0.21). Source reconstruction of gamma power revealed two distinct sources in parieto-occipital cortex (Figure 2E and 2F), which did not differ between percepts (*p*>0.05, permutation test).

We tested the causal role of this interhemispheric gamma-band coherence increase by driving both hemispheres with either in-phase (0° phase difference) (Figures 1C and S1; Text S1) or anti-phase (180° phase difference) tACS for 20 minutes in each session to modulate functional coupling and study subsequent perceptual alterations.

### 40 Hz tACS Modulates Ambiguous Motion Perception

**Figure 3:**
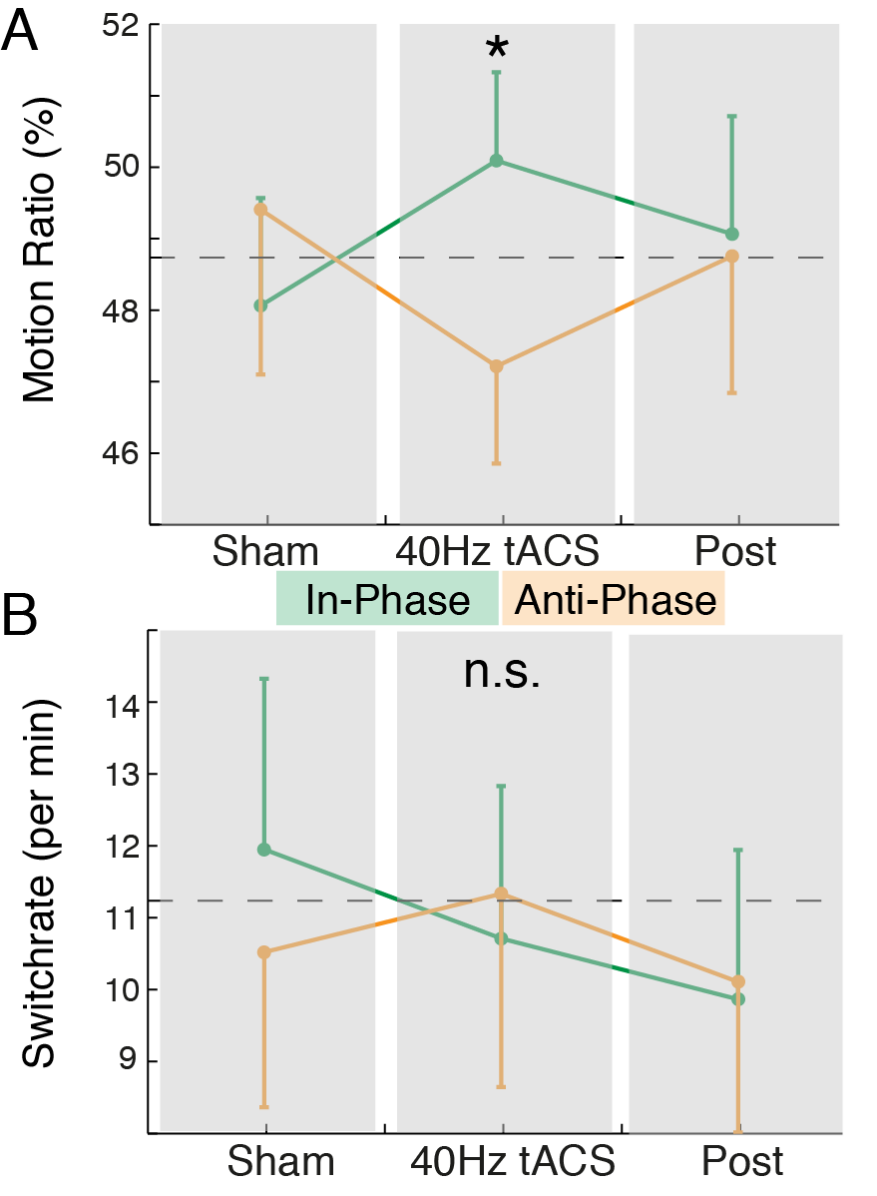
Behavioral results. (A) The MR modulation during the in- (green) and anti-phase (orange) session. The dashed black line depicts the average sham baseline (mean ± SEM). The star indicates the significant difference as revealed by a two-way RM-ANOVA with planned contrasts (Data S1; Table S1). (B) The switch rate across sessions and conditions (same conventions as in (A)).

We assessed the mean duration of horizontal and vertical motion perception by means of a motion ratio (MR  =  time_horizontal_/time_total_) (Table S1) and found a nearly balanced distribution during sham (MR: 48.7%±1.9%, mean ± standard error of the mean [SEM]) (Figure 3A). Our results revealed a significant increase of perceived horizontal motion during in-phase stimulation (+1.4%±1.9%; change to common baseline) (Figure 3A, dashed line) as compared to anti-phase stimulation (−1.5%±1.5%; F_1,13_ = 5.17, *p*<0.05, Cohen's d = 0.59; planned contrasts). This difference was not significant during the post condition (F_1,13_ = 2.70, *p* = 0.12; d = 0.05) (Table S1). No significant results were found for the switch rate (reversals per minute) (Figure 3B; all *p*>0.1). Taken together, the behavioral results suggest that the targeted manipulation of the interhemispheric phase difference biased the conscious experience of apparent motion.

### Phase- and Frequency-Specific Stimulation Effects on Interhemispheric Coherence

**Figure 4:**
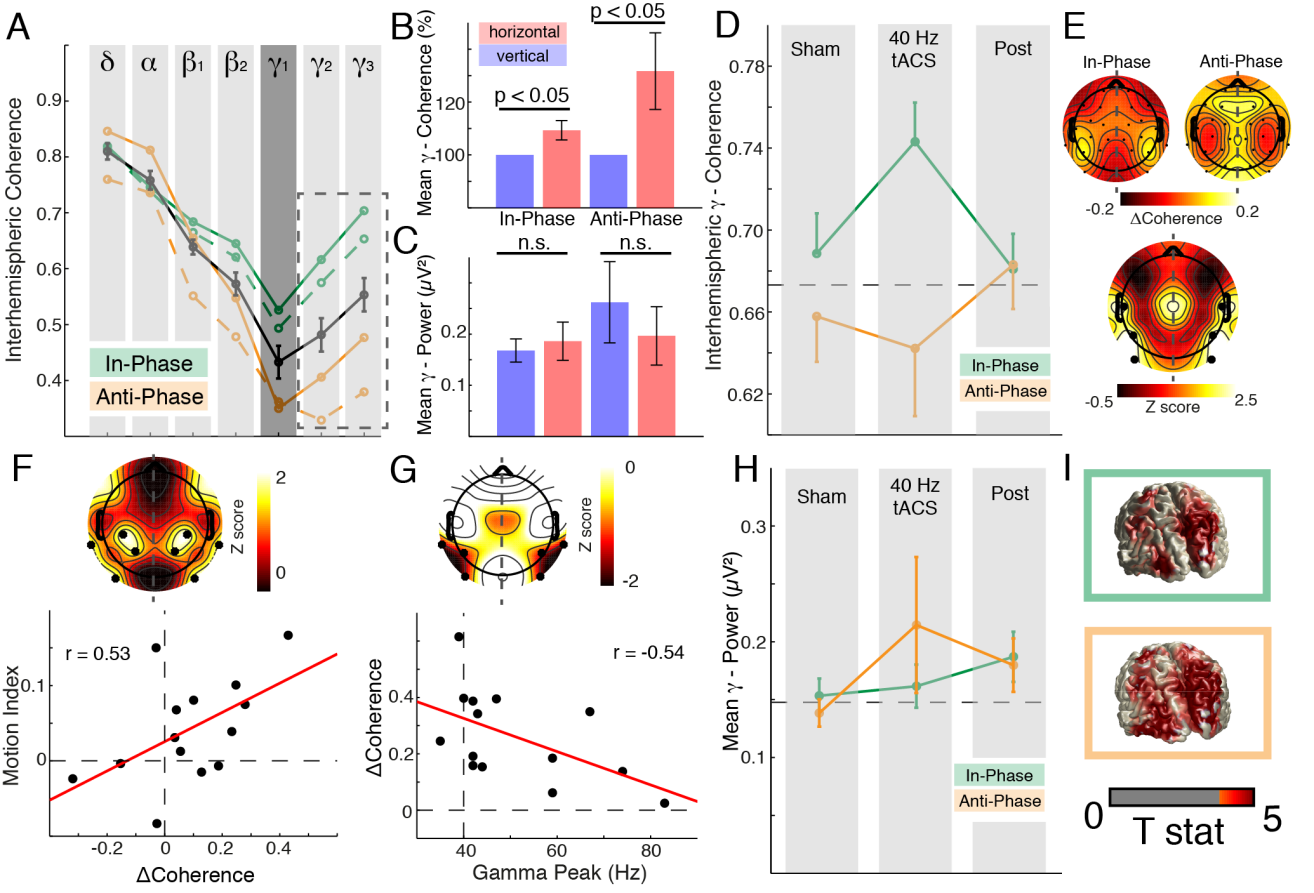
Modulation of interhemispheric coherence during stimulation. (A) Grand mean coherence average over four posterior channel pairs: The solid black line depicts average coherence during sham (mean ± SEM). Notch filtering in the γ_1_-band was applied to all conditions (dark-grey shaded, estimates were obtained after spectral smoothing and not used for statistical analyses; dashed box highlights utilized frequency bands). Dashed lines indicate vertical motion percepts; solid lines depict horizontal percepts. See also Data S1. (B) Relative γ_2/3_-coherence increase during the horizontal percept (with respect to the vertical percept) revealed that the binding-related coherence increase was still present during tACS (same conventions as in Figure 2C). (C) Absolute γ_2/3_-power values indicated that coherence changes were not related to gamma-power changes. (D) Coherence change over time (pair of interest). The black dashed line indicated the mean sham value. See also Data S1. (E) Spatial distribution for the coherence modulation. Upper left: In-phase stimulation. Upper right: Anti-phase stimulation. Lower: The Z-score map indicates a significant difference between in- and anti-phasic tACS located over parieto-occipital electrode pairs. Dots highlight the significant cluster. (F) Cluster-based permuted correlation analysis: A baseline corrected motion index (MI)  =  (MR_InPhase_ − MR_ShamIn_) − (MR_AntiPhase_ − MR_ShamAnti_) was correlated with the baseline corrected ΔCoherence of γ_2_-coherence values (Coh_In-Phase_ − Coh_Anti-Phase_). Upper panel: The black dots in the topography depict the significant cluster (*p* = 0.008). Lower panel: Regression line fitted through the mean coherence values as obtained from the cluster test. Dots depict individual subjects. Solid red line depicts the linear regression. (G) Cluster-based permuted correlation analysis indicated that subjects with an individual gamma coherence peak close to the stimulation frequency (40 Hz) exhibit the largest coherence modulation (*p* = 0.049). (H) Mean gamma power change over time (Data S1). (I) Source reconstruction of gamma activity during in- (green) and anti-phase (orange) stimulation as compared to baseline.

All analyses for the SAM were conducted without the gamma_1_-range (spectral estimates in Figure 4A were obtained after spectral smoothing) to remove the 40 Hz stimulation artifacts. In order to assess whether stimulation selectively affected the gamma-band, grand average interhemispheric coherence values (Figure 4A) were submitted to a two-way repeated measures analysis of variance (RM-ANOVA) (*session*: sham, in-, and anti-phase stimulation; *frequency*: six bands). We report Greenhouse-Geisser corrected values in case of violation of sphericity. A significant interaction of *session × frequency* (F_1.72,22.38_  = 20.5, *p*<0.005; *session:* F_1,13_ = 40.7, *p*<0.005; *frequency:* F_1.56,20.22_  = 52.3, *p*<0.005) indicated a frequency-specific modulation of interhemispheric coherence. Importantly, the increase in gamma-coherence during horizontal motion perception was present in both tACS conditions (Figure 4B; in-phase: +9.3%±3.7%; t_13_ = −2.55, *p*<0.05; anti-phase: +31.7%±14.5%; t_13_ = −2.18, *p*<0.05), without any changes in absolute gamma-band power (Figure 4C; in-phase: t_13_ = −0.55, *p* = 0.59; anti-phase: t_13_ = 1.20, *p* = 0.25). Importantly, a two-way RM-ANOVA with factors *session* (in- and anti-phase) and *percept* (vertical and horizontal) calculated on absolute coherence values (Figure 4A) indicated that the *percept* (F_1,13_ = 8.86, *p*<0.05) related increase in gamma coherence was present in both *sessions* (F_1,13_ = 52.69, *p*<0.005) and was not inflated when absolute coherence values were lower during anti-phase stimulation (interaction: F_1,13_ = 0.81, *p* = 0.38).

In addition, source reconstruction revealed that gamma-band power was again confined to parieto-occipital areas and did not differ between different percepts or stimulation conditions (all *p*-values >0.05; cluster test) (Figure 4I).

Figure 4A indicated that the coherence modulation was confined to the gamma-band. Thus, we analyzed the gamma coherence change over the three conditions with cluster-based permutation statistics and found a significant difference between in- and anti-phase stimulation over stimulated parieto-occipital cortex (cluster test: *p* = 0.006) (Figure 4E), while no differences where present between both sham or post conditions (cluster tests: all *p*>0.05). The modulation at the channel pair of interest (Figure 2A) highlights the difference between the increase during in-phase stimulation (+0.07±0.02; change to common baseline) (Figure 4D, dashed line) and the decrease during anti-phase stimulation (−0.03±0.03; effect size: d = 1.00). This effect was not present between both sham or post conditions (sham: d = 0.39; post: d = −0.03).

Interestingly, the coherence modulation was strongest for subjects with an individual gamma coherence peak frequency close to 40 Hz (Figure 4G; cluster test: *p* = 0.049). The mean gamma peak frequency did not differ between sessions (t_13_ = 0.17, *p* = 0.87; Peak_InPhase_: 51.14 Hz±3.9 Hz; Peak_AntiPhase_: 50.14 Hz±4.02 Hz). Importantly, the observed coherence effect was independent from any power changes (Figure 4H and 4I; all *p*-values >0.2). Taken together, these results indicate that 40 Hz tACS successfully modulated interhemispheric phase synchrony and behavioral outcome, depending on the chosen electrode montage and the subsequently induced phase shifts. The individual percept was influenced by (i) the absolute coherence values (Figure 4A and 4F) and (ii) the relative coherence difference between the vertical and the horizontal percept (Figures 2B, 2C, and 4B).

### Outlasting Coherence Effects Are Related to Phase Differences of Stimulation

**Figure 5:**
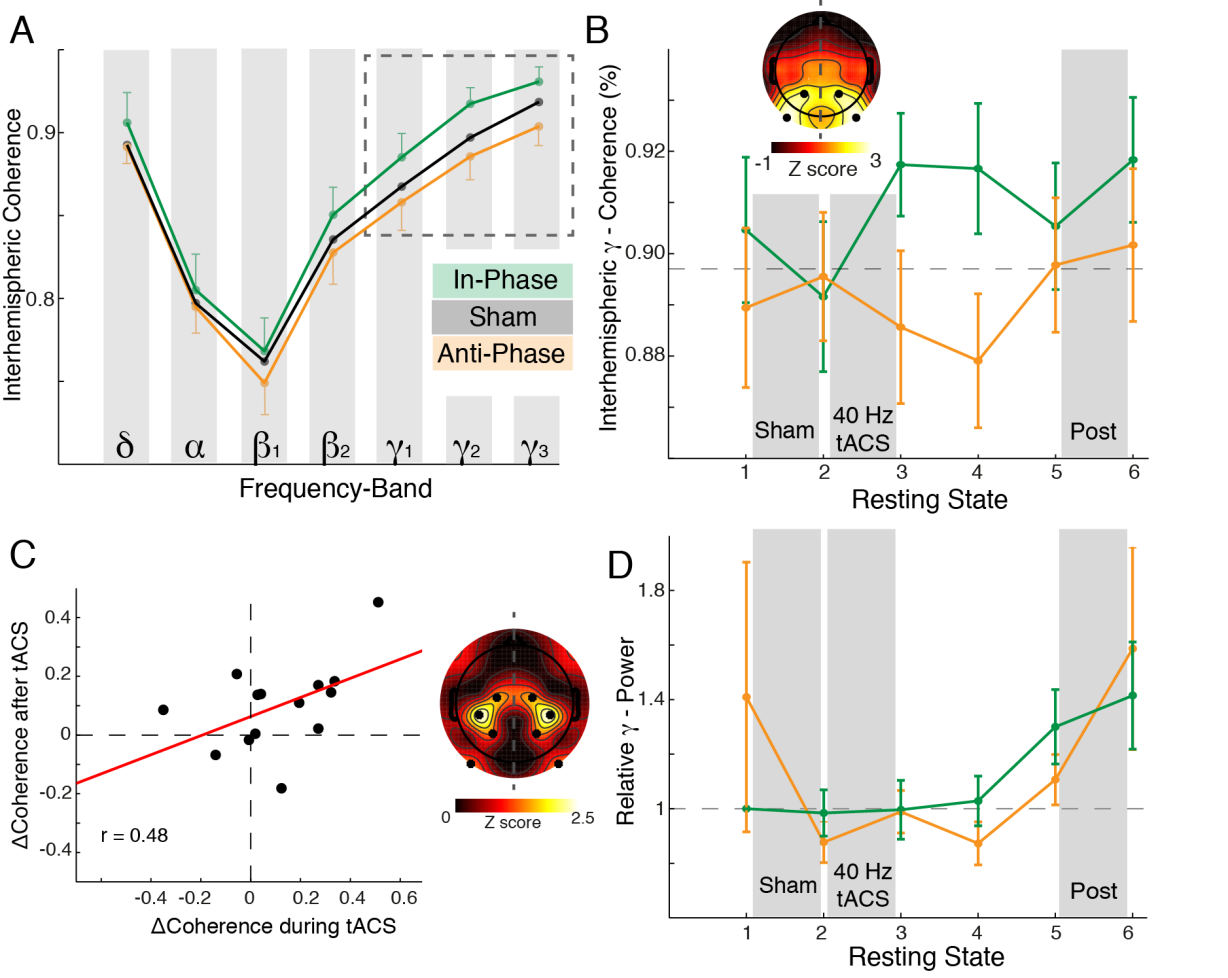
Outlasting coherence effects are frequency and polarity specific. (A) Coherence spectra during RS1 (black), after in-phase stimulation (RS3, green), and after anti-phase stimulation (RS3, orange) are depicted. For further analyses, the complete gamma-band range (γ_1–3_, dashed box) was used. (B) Time course of gamma-band coherence (relative to RS1 during the in-phase session) over six resting state intervals (RS1–6; Data S1). The topography highlights a significant difference after stimulation (RS3) between in-phase and anti-phase stimulation. The cluster had its maximum over parieto-occipital areas (*p* = 0.039; dots highlight the significant cluster). (C) Cluster-based permuted correlation analysis indicated a significant positive correlation between the γ_2_-coherence modulation during and the γ_1,2_-coherence modulation after stimulation (*p* = 0.026). Same conventions as in Figure 4F, all values were baseline corrected. Black dots in topography highlight the significant cluster. (D) Gamma power time course (relative to RS1 of the in-phase session; Data S1).

Entrainment would require that effects on the phase of the ongoing activity should outlast stimulation offset [23]. We found significantly modulated gamma coherence values after *stimulation* (sham, in-, anti-phase stimulation; RM-ANOVA: F_2,26_ = 3.84, *p*<0.05) (Figure 5A). Specifically, we found a significant gamma-band coherence increase following in-phase stimulation (RS3) (Figure 5B) as compared to anti-phase stimulation (t_13_ = 2.62, *p*<0.05; effect size: d = 0.67). We assessed the time course of gamma-band coherence across the six resting state intervals (*time*) with in- and anti-phase stimulation (*session*) with a two-way RM-ANOVA (Figure 5B), and found a significant interaction of *session × time* (F_3.13,40.65_ = 3.08, *p*<0.05; *session*: F_1,13_ = 3.73, *p* = 0.08; *time:* F_3.76,48.92_ = 1.21, *p* = 0.32), indicating that coherence modulation did outlast stimulation offset. This effect was confined to the stimulated parieto-occipital cortical areas (cluster test: *p* = 0.039) (Figure 5B, inset). Importantly, the coherence modulation after stimulation (RS3) was independent from any changes in spectral gamma power (Figure 5D; t_13_ = 0.07, *p* = 0.94). Additionally, we found a significant correlation between coherence modulation during stimulation and the outlasting changes after stimulation (cluster test: *p* = 0.026) (Figure 5C). These findings imply a direct relationship between effects during and after stimulation. Outlasting effects ceased after approximately 20 minutes (Figure 5B).

### Electrophysiological Changes Index Behavioral Alterations

Our results indicate that the coherence change during stimulation was positively correlated with the altered MR (cluster test: *p* = 0.008) (Figure 4F), suggesting that increased interhemispheric coherence sustained the horizontal percept. Interestingly, the interhemispheric coherence and behavioral performance returned to baseline values before the post session (RS5) (Figures 3A and 5B). Those findings demonstrate that exogenously induced transient shifts in interhemispheric coherence selectively modulate perception of ambiguous motion.

### Entrainment of Interhemispheric Phase Synchrony by tACS

**Figure 6:**
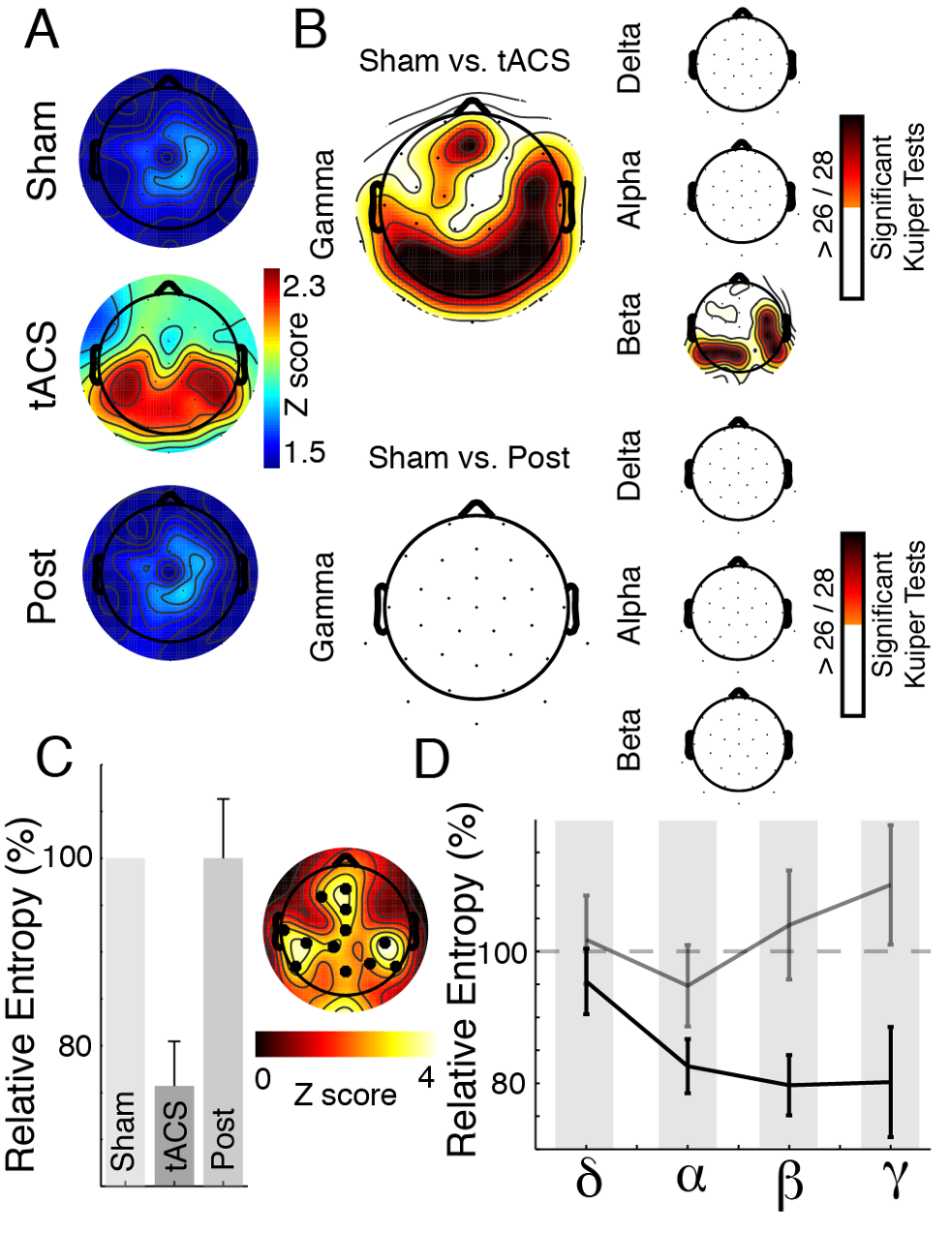
Signatures of gamma-band entrainment by tACS. (A) Results of Rao's spacing test, indicating an increase in non-uniform gamma phase distributions over parieto-occipital cortical areas during tACS. (B) Left: Results of Kuiper's test for unequal distribution of instantaneous phase angles in the gamma-band between sham/stimulation and sham/post, indicate a parieto-occipital maximum (statistical significance is assumed when >26 tests reach *p*<0.0016). Right: The same analysis applied to delta, alpha, and beta-bands. (C) Cluster analysis revealed a significant entropy decrease under parieto-occipital electrodes during stimulation as compared to sham (*p* = 0.004), but not when sham was compared to the post condition (*p*>0.05). A comparison of stimulation and post confirmed this difference (cluster test: *p* = 0.002). See also Data S1. (D) Relative entropy change at a posterior midline electrode during stimulation (dark grey line) and post (light grey line) indicated that the entropy decrease is not confined to the gamma-band (Data S1).

We assessed the phase of the ongoing gamma-band activity during the zero crossing of the external sine wave (every 30 cycles) for four distinct frequency bands (δ, α, β_1/2_, γ_2/3_). For sham and post conditions, a dummy marker was inserted to mimic the tACS trigger events. We tested whether the distribution of instantaneous gamma-band phase angles was non-uniform with Rao's spacing test (Figure 6A) and assessed different distributions with Kuiper's tests. Based on a binomial distribution (*p*<0.0125; Bonferroni-corrected for four frequency bands), we assumed statistical significance at group level when >26 out of 28 comparisons were significant (Kuiper tests: *p*<0.0016 Bonferroni-corrected for 31 electrodes). The same analysis applied to the theta/delta, alpha, and beta-bands, indicated that the effect was most prominent in the gamma frequency range and to a lesser degree still present in the beta-band (Figure 6B).

The continuous visual stimulus presentation restrained us from an event-related analysis such as inter-trial coherence (ITC) and the removal of the γ_1_-band impeded the analysis of a direct interaction between the ongoing activity and the externally applied sine wave. Thus, we analyzed the Shannon entropy across all spectral estimates as a surrogate marker for network dynamics and neuronal entrainment [24].

As shown previously [25],[26], tACS leads to more regular network dynamics and should therefore induce a subsequent entropy decrease. We found that the entropy was significantly reduced during stimulation as compared to sham over parieto-occipital electrodes (cluster test: *p* = 0.004) (Figure 6C). Entropy values returned to baseline during post (cluster test: *p*>0.05). We observed no differences between in- and anti-phase stimulation (cluster test: *p*>0.05). Figure 6D depicts the frequency-specific entropy decrease for both *comparisons* (stimulation/sham and post/sham), with a pronounced decrease in the beta-/gamma-range. These results demonstrated that the entropy decrease was stimulation-specific and indicated that tACS modulated network activity across several temporal scales (Figure 6D). Taken together, the biased phase relationship in gamma-band and more regular network dynamics strongly support entrainment as the putative mechanism of action of tACS [9].

### Entrainment of Oscillatory Gamma-Band Signatures Modulates Alpha Power

The present findings demonstrate that 40 Hz tACS selectively modulates phase relationships in the gamma band (Figures 4A, 4D, 6A, and 6B), without any concurrent gamma power changes (Figure 4C, 4H, and 4I). In light of the entropy decrease across several temporal scales, we further evaluated their physiological interactions [27].

**Figure 7:**
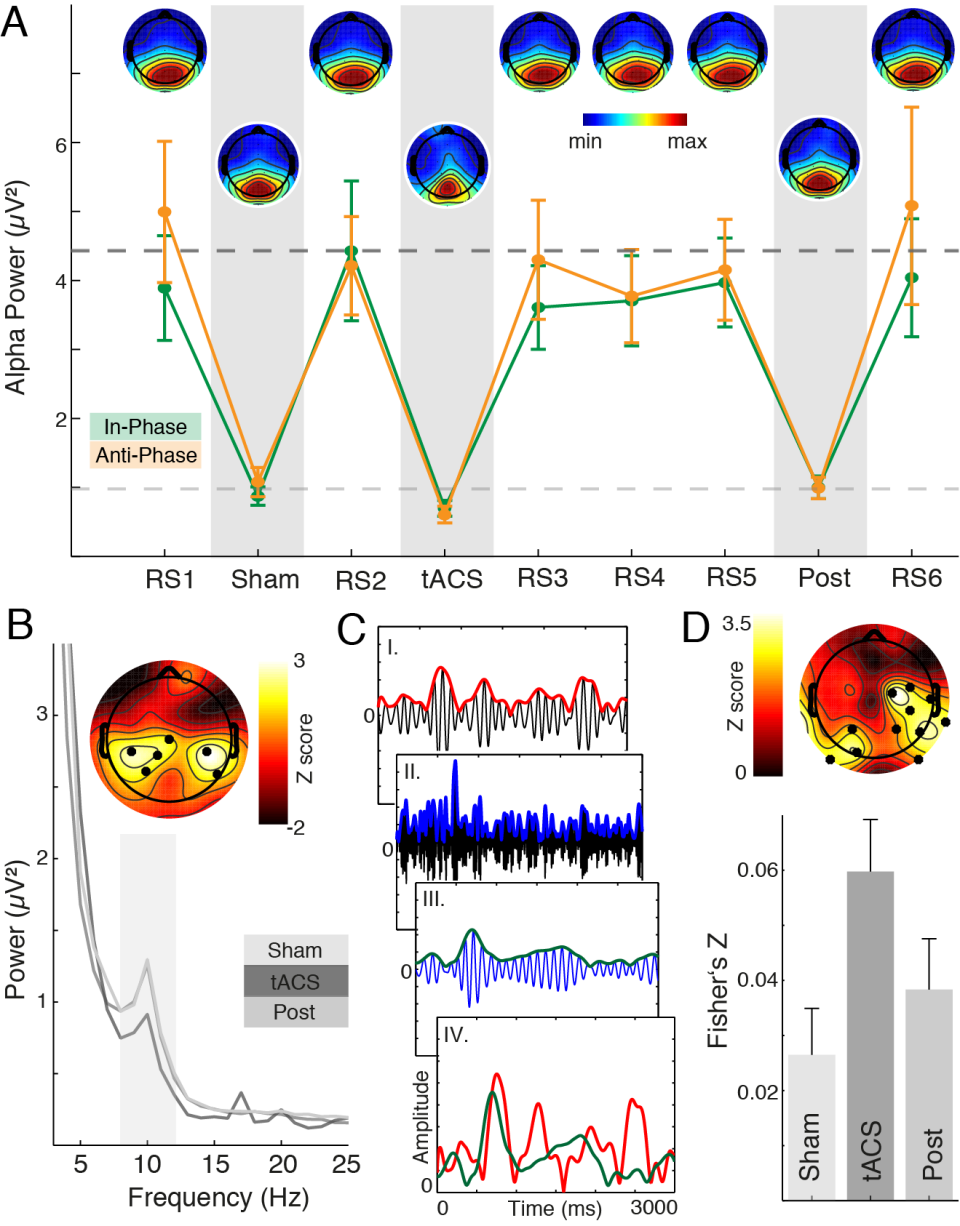
40 Hz tACS modulates alpha oscillations. (A) Mean alpha power (8–12 Hz) over time at lateral parieto-occipital electrodes. Visual stimulation induced a prominent alpha decrease, thus, topographies over time were highly similar. Note that the topography during tACS was less attenuated over lateral EEG sensors (corresponding to (B), upper panel; Data S1). (B) The topography and spectrum depict the significantly reduced alpha power over lateral parieto-occipital areas (dots depict significant cluster, *p* = 0.014; grey shaded area depicts 8–12 Hz range). (C) Schematic amplitude-envelope correlation analysis: I. 8–12 Hz band-pass filtered signal (black) and the corresponding envelope (red). II. Band-pass filtered signal in the γ_2_-range (black) and the corresponding envelope (blue). III. The gamma envelope (blue) was filtered in the alpha range and then its envelope (green) extracted. IV. Superposition of alpha amplitude (red) and gamma envelope (green). (D) Upper: The Z-score map located the main difference between sham and tACS conditions to lateral parieto-occipital sensors. Lower: Relative Fisher-Z-transformed correlation values highlight the transient increase in alpha-gamma amplitude correlations during tACS. See also Data S1.

First, we analyzed the mean alpha power over time (8–12 Hz) (Figure 7A), revealing a prominent alpha decrease during the visual task as compared to fixation (t_13_ = 28.6, *p*<0.0005; −73.5%±2.6%). We assessed the average alpha power by means of a three-way RM-ANOVA and found that alpha power was significantly modulated across *conditions* (sham/stimulation/post; F_1.57,20.44_ = 8.13, *p*<0.005); however, no *session* (in-/anti-phase) or *percept* (horizontal/vertical) related effects were found (*session*: F_1,13_ = 0.20, *p* = 0.67; *percept*: F_1,13_ = 0.08, *p* = 0.79; all interactions: *p*>0.1). Secondly, we assessed the regional specificity of this power decrease and found a significant reduction over lateral parieto-occipital regions in the alpha range during tACS (cluster test: *p* = 0.014; Figure 7B; effect size: d = 1.45).

Given the physiological antagonistic role of alpha and gamma oscillations in the parieto-occipital cortex [27], we investigated cross-frequency interactions between alpha and gamma oscillations. Since no effects on the alpha phase coupling were observed (Figures 4A and 6B), we focused on envelope interactions. We calculated correlations (Pearson linear correlation and Fisher z-transformation) between the alpha-amplitude and the gamma_2_-envelope (Figure 7C). We found a significant cluster when comparing sham and stimulation conditions over lateral parieto-occipital sensors (cluster test: *p* = 0.005; Figure 7D). No differences were present when sham and post conditions were compared (cluster test: *p*>0.05). In addition, we found no differences between in- and anti-phase stimulation (cluster test: *p*>0.05). These observations highlight the physiologic interaction between alpha and gamma oscillations: Entrainment of gamma oscillations promoted a secondary alpha power decrease through enhanced cross-frequency interactions as enforced by the external 40 Hz driving source.

### Control Analyses

The phase-specific tACS effect on interhemispheric coherence was robust across several control analyses. First, magnitude squared coherence values can be affected by changes in amplitude correlation. Thus, we repeated all central analyses based on the phase-locking value [28], which is independent of the amplitude of a given signal. We confirmed the increase in gamma-band phase-locking during horizontal motion perception (116.3%±5.6%; t_13_ = −2.93, *p*<0.05), as well as the phase-specific significant PLV modulation during stimulation (t_13_ = 2.53, *p*<0.05; in-phase: 0.08±0.02, anti-phase: −0.01±0.04). Importantly, the PLV modulation after stimulation offset also confirmed all previous analyses (t_13_ = 3.14, *p*<0.05; in-phase: 0.6%±0.3%, anti-phase: −1.1%±0.6%).

Second, EEG studies investigating gamma-band power can be contaminated by microsaccade artifacts [29]. We therefore analyzed eye-tracking data with respect to fixation and occurrence of microsaccades [30] to exclude potential confounds of eye movements on the observed coherence modulation. We found that subjects reliably fixated during both *sessions* and all *conditions*, independent of their *percept* (three-way RM-ANOVA: all factors and interactions *p*>0.05) (Figure S2). The influence of microsaccades was also assessed in a three-way ANOVA, indicating that the mean number of microsaccades per seconds did not vary between sessions, conditions, or percepts (three-way RM-ANOVA: all factors and interactions *p*>0.05) (Figure S3).

## Discussion

Our results demonstrate the phase-specific modulation of perceptually relevant interhemispheric gamma-band coherence by 40 Hz tACS. Exogenously enhanced functional coupling facilitated the emergence of the horizontal motion percept, whereas the selective impairment reinforced vertical motion perception. Importantly, the induced coherence effects during stimulation did outlast stimulation offset, and thus imply the selective entrainment of oscillatory gamma-band activity. In addition, our results also suggest that rhythmic external stimulation shaped functional network architecture across multiple temporal scales and subsequently reduced oscillatory alpha power through enhanced cross-frequency interactions. This approach offers a unique opportunity to selectively modulate synchronization processes in large-scale neuronal networks in a frequency-specific manner.

### Long-Range Synchronization Mediates Cortical Information Flow

Cortical information flow between distant regions is dynamically established by selective phase synchronization [2],[6] and has been demonstrated for a variety of cognitive functions [3]. Especially, synchronization in the gamma-band has been suggested to constitute a fundamental mechanism for feature integration in the brain [1] and might facilitate the emergence of a stable percept of ambiguous stimuli [20],[31]. In particular, interhemispheric gamma-band coupling might play a crucial role for feature integration across both visual hemifields [7]. However, a number of pitfalls hamper the analysis of long-range synchronization in human EEG studies.

Especially, volume spread in the cortical tissue constitutes a severe constraint for the interpretation of human M/EEG data at sensor level [3]. Here, we utilized source reconstruction to highlight two distinct oscillatory gamma sources in the parieto-occipital cortex. The absence of any power differences between conditions or percepts reinforced our conclusion that interhemispheric coherence changes were a direct consequence of altered phase coupling and could not be attributed to differences in source configuration. Novel methods for coupling analysis at source level have successfully been introduced in the past [32]. However, these techniques ideally require the use of many more electrodes than employed in this study. Only relatively few electrodes have been utilized here, to avoid amplitude clipping of adjacent EEG electrodes during tACS, since stimulation currents exceed the usual recording range of EEG amplifiers by several orders of magnitude [9].

Previously, several connectivity measures have been introduced, which suppress coherent activity at 0° phase difference [33],[34], thus minimizing the effect of volume conduction at the expense of ignoring physiologic synchronized neuronal activity with 0° phase difference [35]. Here, we reconstructed oscillatory gamma power at source level to rule out that changes in oscillatory power may account for any of the observed coherence or phase-locking differences as observed at scalp level. We utilized sham stimulation as a baseline to control for these effects, since it is unlikely that volume conduction changes as a function of condition. Likewise, the vertical percept served as a baseline for the horizontal percept. Importantly, the directionality of behavioral and electrophysiological effects was directly related to opposite stimulation polarities. Hence, we assume that the key findings of this study were not affected by current methodological limitations.

### Entrainment of Perceptually Relevant Brain Oscillations

Neocortical spike activity is directly controlled by weak electric fields generated by the cortex itself [36]. Recently, it has been shown that externally applied weak electric fields may mimic endogenous fields and therefore may modulate the temporal structure of large-scale neuronal networks [14],[25] and synchronize spiking activity [36] to different driving frequencies in a phase-specific manner [13].

Entrainment of perceptually relevant brain oscillations in humans has been demonstrated for repetitive transcranial magnetic stimulation (rTMS) [37] and tACS [9], but the exact mechanisms of action are still largely unknown. The direct interaction of a cortical oscillator and a rhythmic external source by synchronization has been suggested to constitute a key mechanism for entrainment [8]. Computational models indicated that the intrinsic network frequency is ideally suited to entrain the network [25]. Nonetheless, stimulation at adjacent frequencies with higher stimulation intensities can also entrain the network sufficiently [25],[26] and might explain the observed effects of the 40 Hz stimulation on the γ_2/3_-range. Importantly, the strongest coherence modulation was observed in subjects with an intrinsic gamma peak frequency close to 40 Hz, emphasizing the need for frequency-matched stimulation protocols in future studies [38].

Previously, the assessment of neuronal activity during stimulation has been hampered by the difficulty to remove stimulation artifacts in concurrent EEG recordings. Recently, a novel approach for artifact rejection has been introduced to remove 10 Hz tACS artifacts [9]. However, its applicability is limited to lower stimulation frequencies, since the jitter in the exact tACS trigger location, caused by an internal-clock-mismatch between tACS and EEG devices, is amplified during 40 Hz tACS. Therefore, a notch-filter was applied to remove the stimulation artifact, a procedure that is commonly used for line noise removal [39]. Hence, we focused the analysis during stimulation on effects in adjacent frequency bands. Importantly, immediate stimulation effects on the phase of the ongoing activity were still present after stimulation offset, thus, making a successful gamma modulation with 40 Hz tACS highly likely. Current limitations concerning the artifact removal might be overcome with synchronized tACS-EEG systems and improved artifact rejection algorithms [9]. In addition, the combination of tACS with different imaging modalities, such as fMRI [40] or magnetoencephalography (MEG) [41] might extend our understanding of the physiological efficacy of tACS.

The modulation of long-range functional connectivity has been demonstrated for repetitive transcranial magnetic stimulation (rTMS) [42] and tACS [15]. However, none of the above studies presented conclusive behavioral and electrophysiological evidence for a successful modulation of perceptually relevant long-range synchronization.

The study by Strüber and colleagues [18] also employed the SAM paradigm. However, the authors found a divergent pattern of results. At the behavioral level, their results suggested that only anti-phasic stimulation at 40 Hz effectively modulated the conscious experience of apparent motion. Here we replicated the behavioral key finding, i.e., that anti-phase stimulation at 40 Hz introduces a bias to vertical motion. We extended the behavioral findings by demonstrating that a HD-tACS electrode montage may lead to a more focal in-phase stimulation (Figure S1), which biases the SAM perception to horizontal motion. In contrast, the in-phase montage used by Strüber and colleagues mainly targeted the occipital pole and resulted in no perceptual bias (Figure S1). Taken together, both studies suggest that 40 Hz tACS biases apparent motion perception, irrespective of SAM stimulus parameters or presentation (foveal or parafoveal) (Table S2) [43]. At the electrophysiological level, Strüber and coworkers reported that anti-phase stimulation resulted in increased interhemispheric coherence after stimulation, while we found the opposite pattern of results, i.e., in-phase stimulation enhanced synchronization, anti-phase stimulation impaired functional coupling. Strüber and colleagues had interpreted this apparent contradiction as functional decoupling, i.e., that two signals with opposite polarities still might be highly coherent as long as the phase shift remains constant. Given the differences in study design, stimulus presentation, and tACS settings (for a detailed overview please see Table S2), we assume that the divergent patterns might result from the fact that even slight variations in stimulation intensity or electrode montage might lead to opposite network effects [44]. In addition, divergent results might also be explained by the cortical network state dependence of tACS effects [14],[38]. These findings highlight the need for well-controlled tACS protocols, which should ideally be based on computational models and electric field predictions [25],[45].

In this study, we based our hypothesis on phase-specific electric field predictions (Figure S1) and subsequently presented evidence for the selective modulation of perceptually relevant interhemispheric gamma synchronization. Crucially, coherence effects, as induced during stimulation, outlasted the offset by approximately 20 minutes.

It has been argued that the low spatial specificity might constitute a severe limitation of tACS [46]. However, this characteristic might prove beneficial for the modulation of large-scale networks. Here we utilized bilateral 4×1 ring electrode montages [17] to selectively stimulate regions of extrastriate visual cortex involved in motion perception (Figure S1) [21],[47]. In the future, multi-channel stimulators and additional modeling work [16] will hopefully allow more focal stimulation settings.

Interestingly, we found that network dynamics across multiple temporal scales become more regular during external rhythmic stimulation. In particular, our results reveal that alpha power was selectively reduced after entrainment of gamma signatures. However, effects on phase coupling were mainly observed in the gamma-band. Our results support the idea that tACS effects on the phase of the ongoing activity are frequency-band specific [46], but ancillary effects might not be constrained to a single frequency-band [11]. At present, it remains unclear whether neuronal entrainment is the only mechanism contributing to the coherence effects or whether mechanisms of neural plasticity are equally important [48]. In absence of effects on alpha phase coupling, it is unlikely that entrainment induced the transient alpha power decrease. Our results rather imply that entrainment of gamma-band activity influenced the physiological antagonistic alpha-gamma interplay in the parieto-occipital cortex [27],[39]. Conversely, the alpha power decrease might serve as a surrogate marker for the selective entrainment of gamma band signatures, since this modulation resembles a physiologic antagonistic response to pronounced gamma band activity.

### Physiological Efficacy of 40 Hz tACS

A number of recent studies have demonstrated that 40 Hz tACS modulates cognitive processing, e.g., enhances fluid intelligence [49], induces lucidity in dreams [50], or modulates the conscious experience of apparent motion [18]. So far, most studies utilized 40 Hz stimulation to entrain the gamma-band, even though some evidence suggested that 60 Hz tACS might be more effective than 40 Hz tACS [51]. However, results by Voss and colleagues [50] indicated that the efficacy of tACS at 25 Hz and 40 Hz might be similar. Currently, it is unclear whether there are distinct sub-bands within the gamma-band [52] and, furthermore, whether they can selectively be entrained with tACS. Previous tACS-EEG studies suggested very narrow-banded effects of tACS [9],[50], while our present results indicate that 40 Hz stimulation modulated the gamma-band in a broad frequency-range. Importantly, we found that the effects of 40 Hz tACS were mainly confined to the gamma-band, indicating that tACS operates within canonical frequency boundaries constituting the rhythmic brain architecture [53]. These findings imply that tACS might be a powerful tool to assess causal contributions of certain frequency-bands to distinct cognitive processes. Given that we observed clear cross-frequency interactions operating within physiologic boundaries, we urge caution when interpreting tACS effects in absence of electrophysiological recordings [54].

Interestingly, our data also indicated that subjects with a gamma coherence peak close to 40 Hz exhibited the strongest coherence modulation during 40 Hz tACS (Figure 4G). Thus, previously observed effects might be contorted due to a large intersubject variability. These findings further highlight the need for a rational design of tACS protocols [55].

### The Role of Cortical Network States for Perception

Recently, it has become evident that cortical dynamics across multiple spatiotemporal scales influence conscious perception [3],[56]. Multi-stable phenomena have been related to changes in oscillatory activity in large-scale neuronal networks and might therefore reflect the periodical and constant reevaluation of sensory input [57]. Crucially, it has been shown that both, local ongoing activity [58] and interregional network activity [4], influence subsequent perception. Bistable perception is ideally suited to study underlying network activity, since identical sensory input can lead to distinct percepts, depending on the current network state [4].

Previously, perception of the SAM has been linked to different spectral features. In particular, it has been demonstrated that frontal gamma power increments [59] together with parieto-occipital alpha power decrements [60] precede a perceptual switch. Importantly, the subjects' perceptual bias was influenced by the level of interhemispheric gamma-band coherence (Figure 2C) [20] and the individual percept was enforced by selectively modulating coherence levels by tACS. Frontal gamma-band increments might trigger changes in parieto-occipital networks [61] and thereby influence perception. Our data suggest that the selective entrainment of interhemispheric gamma-band synchrony might mimic the physiologic mechanism of top-down controlled percept reversals. A similar mechanism has previously been demonstrated for bottom-up processes with lower level neuron populations entraining rhythmic patterns in higher cortical areas in a feed-forward fashion [62].

Complementary coupling analyses in source space employing human M/EEG [3] will be necessary to determine whether top-down control of the SAM is associated with the selective entrainment of interhemispheric phase synchrony.

Interestingly, decreased parieto-occipital alpha activity has been linked to a destabilization of the SAM [60]. In our study, we found a decrease in alpha activity in response to the entrainment of physiologic gamma-band signatures, however, without any accompanying switch rate changes (Figure 3B). This observation indicates that the alpha decrease might actually reflect a secondary process in response to the gamma mediated perceptual reversals, thus, highlighting the antagonistic role of alpha and gamma oscillations [27].

Taken together, our findings strongly support the idea that neuronal interactions across different cortical regions are encoded at multiple temporal scales [56] by selective synchronization between task-relevant areas [3].

### Confounds and Limitations

tACS studies have a seemingly endless search space: Electrode placement, stimulation frequency, intensity, and duration are obvious concerns. Here we based our hypothesis on two recent SAM studies [18],[20] and subsequently reproduced their main findings. We documented the electrophysiological signatures of SAM perception in absence (Figure 2A–2C) and in presence of tACS (Figure 4A and 4B). However, a number of limitations do apply. (i) We only applied tACS for 20 minutes, since it had previously been shown that 20 minutes of 40 Hz tACS effectively modulated apparent motion perception [18]. While 20 minutes of stimulation are well within current safety limits [63], it is unclear how stimulation duration impacts the behavioral and electrophysiological outcome. (ii) Furthermore, our results suggest that stimulation at individual peak frequencies might actually be more efficient (Figure 4G) than using a fixed stimulation frequency. (iii) Another limitation is the preceding sham session. In accordance with previous results [9],[38], we observed outlasting stimulation effects for approximately 20–30 minutes, which impede an inverse procedure. Here we utilized a post condition to validate the sham condition and to control for outlasting behavioral and electrophysiological effects. Throughout the study we did not find any differences between sham and post conditions. Furthermore, the counter-balanced within-subject design in two sessions allowed us to study directionality effects, thus, minimizing concerns that the subjects could distinguish between real and sham stimulation. In fact, it was impossible for subjects to distinguish in- and anti-phase stimulation. (iv) Another clear restriction is the unbalanced electric field distribution (Figure S1). While our in-phase stimulation was very focal, the anti-phase montage led to a more distributed electric field. However, in contrast to the study by Strüber and colleagues [18], both montages targeted the extrastriate visual cortex and modulated the behavioral outcome. We believe that multi-channel stimulators and optimized electric field models will improve the focality of tACS in the future. We expect that a rational design of tACS experiments using frequency-matched and neuro-navigated protocols will improve the efficacy in human tACS studies [55].

So far, the basic physiological principles behind the efficacy of tACS are still largely unknown [46]. Further work in human, animal, and modeling studies will hopefully advance our understanding of tACS and its interactions with neuronal circuits. In the future, complementary modeling approaches may guide individually tailored stimulation protocols and electrode features to overcome current limitations, such as the unclear electric field distribution in the head, the cortical state dependence, and the high intersubject variability.

## Conclusions

In summary, our results demonstrate that interhemispheric gamma-band coherence can be selectively modulated by tACS. In this study, we established a causal role of synchronized gamma band oscillations for feature integration across both hemispheres and confirmed the antagonistic role of alpha and gamma oscillations in the parieto-occipital cortex [27].

Our results demonstrate the ability of tACS to selectively entrain cortical oscillations and add to a growing body of evidence indicating that synchronized oscillatory activity in large-scale neuronal networks is a key mechanism for conscious perception and cognition [3],[56]. Disturbances of synchronized network activity have previously been related to schizophrenia, autism spectrum disorders (ASDs), and Parkinson's disease [64]. In particular, ASD have been associated with impaired feature integration across both hemispheres [65].

Future research might therefore offer the possibility to individually tailor therapeutic interventions by means of non-invasive brain stimulation [66]. In particular, the frequency specificity of tACS makes it an ideal candidate for treatment of rhythmic cortical disturbances, as recently demonstrated for tremor suppression in patients with Parkinson's disease [10].

## Material and Methods

### Participants

In accordance with previous studies [18],[20], 14 healthy volunteers (eight females, six males, mean age: 27.5±6.7 years) were recruited from the University Medical Center in Hamburg, Germany, including two of the authors (RFH, HK). All participants (including participating authors) were blinded towards the stimulation sequence. All subjects were right-handed, reported no history of neurological or psychiatric disease, and were medication-free during the experiments. They all had normal or corrected-to-normal vision. All participants gave written informed consent according to the local ethics committee and the Declaration of Helsinki. This study has been approved by the local Ethics Committee of the Medical Association in Hamburg, Germany (IRB number: PV4335).

### Stimuli and Procedure

The SAM (Figure 1A) was generated with the Psychophysics Toolbox [67] implemented in MatLab (The MathWorks Inc.) and presented on a BenQ XL2420T screen (1,920×1,080, 120 Hz). The display was 60 cm away from the participants. The horizontal dot distance was 5.1°, the vertical 6.9° at a constant dot size of 0.35°. We introduced a shorter horizontal than vertical distance to compensate the vertical bias of equidistant SAMs [19]. Every trial of continuous visual stimulation lasted 1 minute. Participants reported their percept by pressing two different buttons with their right hand. All volunteers participated in two sessions of the experiment carried out within 1 week. After preparation of the tACS and EEG electrodes (see below), participants completed a training session with ambiguous and non-ambiguous trials to familiarize volunteers with the stimulus and to ensure that all participants could reliably track their percepts. On both days all participants completed ten trials during sham, 20 trials during stimulation (in- or anti-phasic tACS), and ten trials during the post condition. Whether participants were stimulated with in- or anti-phase stimulation on the first day was counterbalanced across subjects (Figure 1B, the participating experimenters were blinded whether they received in- or anti-phasic stimulation). A sham condition always preceded electrical stimulation to avoid carry-over effects [38]. Altogether, six resting state epochs, 3 minutes each, were recorded during stable fixation of a central dot to assess outlasting changes.

### Data Acquisition

#### EEG recording

All experiments were conducted with participants seated comfortably in a recliner in a dimly lit, electrically shielded room to avoid line noise interference. EEG and tACS Ag/AgCl electrodes were mounted in a custom-made elastic cap for 128 electrodes (Easycap) prepared with a slightly abrasive electrolyte gel (Abralyt 2000, Easycap). A ground-free EEG recording (no amplitude clipping, impedances <20 kΩ, referenced to the nose tip) was obtained from 31 EEG channels in an equidistant array (inset Figure 2A) using BrainAmp amplifiers (Brain Products GmbH). Signals were sampled at 5,000 Hz, amplified in the range of ±16.384 mV at a resolution of 0.5 µV and stored for offline analyses.

#### tACS

Transcranial stimulation was applied by a battery-operated stimulator (DC-Stimulator Plus, NeuroConn) via ten Ag/AgCl electrodes (12 mm diameter, Easycap) (Figure 1C) [17], resulting in a combined electrode area of approximately 11.3 cm^2^. Electrode placement was chosen in accordance with previous electrical brain stimulation studies targeting the extra-striate visual cortex [18]. The combined impedance of all electrodes was kept below 5 kΩ, as measured by NeuroConn tACS device, using Signa electrolyte gel (Parker Laboratories Inc.) [68]. A sinusoidally alternating current of 1,000 µA (peak-to-peak) was applied at 40 Hz continuously for 20 minutes during each session. A tACS trigger was inserted every 30 cycles during the zero crossing of the external sine wave. The stimulation frequency was chosen in accordance with a recent tACS study, which demonstrated behavioral effects at 40 Hz, but not for 6 Hz or sham stimulation [18]. During sham and real stimulation the current was ramped up over 20 seconds to 1,000 µA, but discontinued during the sham condition. All subjects confirmed that stimulation was acceptable and mainly noticeable during the ramp-in phase. It did not induce painful skin sensations or phosphenes. On debriefing, subjects indicated that they could not distinguish between sham, stimulation at different polarities, and the post session.

#### Eye-tracking data

In order to control for fixation and eye movements, the eye position was recorded in all trials with a monocular eye-tracking system (EyeLink 1000, SR Research) in 12 out of 14 subjects. Eye position was sampled at 500 Hz and stored for offline analysis.

### Data Analysis

#### Behavioral data

The horizontal MR was defined as follows: MR  =  time_(horizontal percept)_/time_(total)_. The first 3 seconds of every trial were excluded and only percept durations >1 second were accepted to ensure the emergence of a stable percept. Redundant button presses were discarded (on average 0.43%±0.58%, mean ± standard deviation [STD]). The switch rate was defined as the mean number of indicated perceptual switches per minute.

#### EEG data

Data analysis was performed using MatLab (The MathWorks Inc), EEGLab) [69], FieldTrip [70], and customized MatLab code. All preprocessing steps were performed with EEGLab. The recorded EEG was filtered between 1 Hz and 100 Hz using two-pass finite element response filters (eegfilt.m function) as implemented in EEGLab and down-sampled to 1,000 Hz. For the analysis of the SAM data, a higher (>6th) order two-pass butterworth notch filter in the range from 35 to 45 Hz was applied to the sham, stimulation, and post measurements to remove the stimulation artifact and to keep results comparable across all conditions. Forward and backward filtering was performed to avoid phase distortions. Note, that sham and post conditions were also analyzed without the notch-filter for Figure 2. Then the data was segmented into 3,000 ms epochs, starting 500 ms after an indicated perceptual switch. Epochs containing excessive noise, saccades, or muscle artifacts were removed after visual inspection. Data acquired during resting states was split into 1-second segments. Segments containing artifacts were excluded after visual inspection.

Spectral estimates for SAM and resting state data were computed by means of a sliding Hanning window (1–35 Hz, 1 Hz steps, 500 ms window) and a multi-taper approach [71]; (36–100 Hz, 1 Hz steps, 250 ms window length, ±10 Hz frequency smoothing, SAM: 59 slepian tapers, resting state [RS]: 19 slepian tapers). Slow frequencies (delta/theta) were pooled and treated as one frequency band given that (a) delta-band activity is likely confounded by the stimulus presentation rate (visual tokens alternated every 250 ms and (b) a recent study already demonstrated that 6 Hz tACS did not impact apparent motion perception [18]. Coherence estimates were calculated for 13 interhemispheric channel pairs (inset Figure 2A) in 1 Hz steps and then averaged across the frequency bands of interest. The increase in interhemispheric coherence was quantified as the relative coherence increase for horizontal over vertical motion perception, since the vertical percept serves as a rigorous baseline that controls for effects such as volume conduction or reference electrode position [20]. The number of data segments per subject was balanced across conditions and percepts to avoid a sample size bias.

Cortical sources of gamma-band activity were reconstructed with a linear beamforming approach [32] as implemented in FieldTrip. A volume conduction model was derived from the MNI template brain, resulting in a three-shell model. The leadfield matrix was calculated using the boundary element method. The source activity at each grid point was estimated by constructing a spatial filter using the leadfield at a given point and the cross-spectral density matrix. For each participant and condition, the cross-spectral density matrix was calculated between all 31 EEG channels, separately for the RS (baseline) and the respective conditions and percepts. Differences were assessed by cluster-based permutation statistics [72].

In order to assess whether network activity was more regular during stimulation, we calculated the Shannon entropy at every channel for every condition on normalized spectral estimates S_x_, i.e., for each frequency the spectra were divided by the sum of all estimates across channel and these quantities were formally treated like probabilities. The entropy H(X) was calculated for four frequency-bands of interest f (δ, α, β_1/2_, γ_2/3_) at every given channel x as:
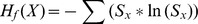



Cross frequency interactions between alpha and gamma were assessed for every channel, condition, and subject separately. In order to calculate the amplitude-envelope-correlation between both, we first band-pass filtered every trial in the alpha and the gamma_2_ range (Figure 7C), similar to the approach, which had been introduced and validated by Voytek and colleagues [39] for 1,000 ms data segments. We also utilized a two-way, zero phase-lag, finite impulse response filter (eegfilt.m function in EEGLab toolbox [69]) to prevent phase distortion. Then Hilbert's transform was applied to extract the amplitude of the alpha (Aα) and the gamma (Aγ) oscillation. Then a second Hilbert transform was used to extract the envelope (Eγ) of the band-pass (8–12 Hz) filtered gamma amplitude. Amplitude-envelope-correlations between Aα and Eγ were calculated for every trial separately. Subsequently, correlation values were Fisher-Z-transformed and averaged.

The instantaneous phase of the ongoing activity was also derived from the Hilbert transformation, after band-pass filtering the signal in different frequency bands as outlined above, and subsequently used for circular statistics.

#### Statistical analyses

In accordance with previous reports [18],[20], we focused our analysis on the electrode pair of interest (Figure 2A, red in inset). If not stated otherwise, we used repeated measures analyses of variance (RM-ANOVAs) with post hoc planned contrasts according to our hypothesis. Data were tested for normality with Lilliefors test and for sphericity with Mauchly's test. Where applicable, Greenhouse-Geisser correction was applied and corrected *p*-values and degrees of freedom are reported. Effect size was quantified by means of Cohen's d. All values reported are mean ± SEM. We confirmed the regional specificity of the observed effects with permutation statistics as implemented in Fieldtrip (Monte Carlo method; 1,000 iterations; *p*<0.05). The cluster approach corrects for the multiple comparison problem [72]. Dependent samples *t*-tests were computed at each sensor pair and for each frequency. Clusters were obtained by summing up *t*-values, which were adjacent in space and frequency below a cluster alpha level of 5%. A permutation distribution was computed by randomly switching condition labels within subjects in each of 1,000 iterations. The permutation *p*-value was obtained by comparing the cluster statistic to the random permutation distribution. The observed clusters were considered independently significant when the sum of *t*-values exceeded 95% of the permutated distribution. Circular statistics were calculated with the CircStat toolbox for MatLab [73]. We utilized Rao's spacing test to assess the non-uniform distribution of instantaneous phase angles, which is advantageous for assessment of multimodal non-uniform distributed data, in order to account for the procedure with two sessions. Differences in non-uniform circular distribution between conditions were assessed with Kuiper's tests.

Cluster-based permuted correlation analyses: In order to link the coherence modulation during stimulation to (i) the change in behavior and (ii) the outlasting coherence effects, we implemented a data-driven cluster-based permutation test based on Pearson's linear correlation coefficient. We calculated a baseline corrected composite measure of the behavioral MR by means of a motion index: MI  =  (MR_InPhase_ − MR_ShamIn_) − (MR_AntiPhase_ − MR_ShamAnti_), resulting in a single value per subject. During each iteration of the permutation test, we compared the coherence difference ΔCoh  =  Coh_InPhase_ − Coh_AntiPhase_ to the difference between both sham conditions and calculated a correlation value per channel pair. We utilized the Monte Carlo method for cluster tests as implemented in Fieldtrip. Therefore, we transformed the resulting correlation coefficients (r-values) into *t*-values using the following formula (*n* =  number of subjects): 
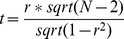



Then clusters were formed based on combining adjacent sensors with *p*-values below a cluster alpha of 20% and summing up *t*-values. A permuted distribution was computed randomly switching condition labels within subjects in each of 1,000 iterations. On every iteration, clusters were again formed based on combining neighboring channels with *t*-values below the cluster alpha. The sum of *t*-values from the largest cluster was added to a permutation distribution. Finally, only clusters spanning at least two adjacent channels with a *p*<0.05 tested against the permutation distribution were considered significant. The same approach was used to compare the coherence modulation in the gamma-band after stimulation offset. However, here we calculated a coherence modulation index per channel pair after stimulation offset, resulting in a channel pair × subject matrix that was used for the correlation analysis. Hence, correlation values were not computed across different channel pairs.

#### Eyetracking data

Data analysis was performed using a previously established algorithm [30] and customized MatLab code. Data within 1 second of a button press was discarded. Fixation was quantified as the average eye position for every subject across all trials, for all sessions, conditions, and both percepts separately. Microsaccades were analyzed by means of a two-step approach: First, data were filtered for saccades <0.05° or >1.6° visual angle and eye blinks. Epochs with less than 80% remaining samples were automatically rejected. Running the algorithm again on the remaining filtered epochs, microsaccades were classified as samples exceeding the average velocity by more than eight standard deviations. They had to have a duration of at least 10 milliseconds and an amplitude between 0.05° and 1° [30]. Vertical and horizontal microsaccade directions were then assessed for every session, condition, and percept direction (vertical and horizontal) separately.

#### Data availability

The dataset used to reach the main conclusions drawn in this study is available in Data S1 and is also deposited in the Dryad Data Repository: http://dx.doi.org/10.5061/dryad.b0r5q [74].

## Supporting Information

Figure S1 **Related to results: electric field simulation.** Electric field simulation for the present study (upper row) and the study by Strüber and colleagues (lower row [18]). Simulations on the left depict in-phase stimulation; simulations on the right depict anti-phase stimulation. Stimulation electrode positions are highlighted on the small panel on a 2D topographic map (same color conventions as in Figure 1C). Electrode placement in the study by Strüber and colleagues was according to the international 10–10 system: In-phase stimulation was delivered over C3, C4, and slightly posterior to O1 and O2, while anti-phase stimulation was delivered over P7, PO7, P8, and PO8. In the present study, the stimulation polarity for the anti-phase session was adjusted to closely match the montage as introduced by Strüber and colleagues. The electric field simulations of the present study indicate that both montages targeted the extrastriate visual cortex and induced subsequent behavioral modulations, with the in-phase setup providing a more focal stimulation. In contrast, the anti-phase montage by Strüber and colleagues also targeted the extrastriate cortex and induced subsequent behavioral alterations, while their in-phase montage targeted mainly the occipital pole and induced no subsequent behavioral modulation. Note that only the relative electric field spread is depicted.
(TIF)

Figure S2 **Related to control analysis: eye data on fixation.** Mean deviation from fixation cross (positioned centrally at 970×540 pixels) for sham, stimulation, and post conditions during the in-phase (upper) and anti-phase (lower) session. Black dots in corners depict possible positions of the SAM. Grey line spacing is 1° visual angle. Red and blue dots depict each subject's mean position during perceived vertical (blue) and horizontal (red) motion.
(TIF)

Figure S3 **Related to control analysis: eye data on microsaccades.** Upper panel: Mean microsaccade rate per second (± STD) for all sessions and conditions during horizontal motion perception. Lower panel: Mean microsaccade rate per second (± STD) for all sessions and conditions during vertical motion perception. Dashed grey lines depict 1.5 microsaccades per seconds.
(TIF)

Table S1 **Related to** **Figure 3:** **planned contrast analysis.** Supplemental behavioral modulation results: Full statistical comparison in a planned contrast analysis according to our hypothesis. In addition, partial eta squared indicates the effects size for all factors. Contrast L1: In- versus anti-phase session; Contrast L2: Sham versus stimulation; Contrast L3: Sham versus post.
(DOCX)

Table S2 **Related to Discussion: comparison of the present study to Strüber and colleagues** [1],[18] **.** Strüber and colleagues [1],[18] were the first to investigate the impact of tACS on ambiguous motion perception. Their results indicated that only anti-phasic stimulation at 40 Hz influenced the conscious experience of apparent motion perception. The authors obtained no significant results for in-phase stimulation at 40 Hz, nor did they find any effects for 6 Hz or sham stimulation. In particular, they demonstrated that anti-phasic 40 Hz tACS increased the amount of perceived vertical motion in the SAM. Here, we replicated their key behavioral finding and adjusted several experimental parameters to demonstrate that interhemispheric coherence could, in fact, be modulated in a desired and predictable fashion. Main differences are outlined in the table above. The most important differences are highlighted in bold type.
(DOCX)

Text S1 **Related to** **Figure 1C:** **electric field simulation.** Supporting information for the modeling of the electric field. Results of the modeling can be found in Figure S1.
(DOCX)

Data S1 **Related to Results.** Excel table containing individual observations underlying summary data presented in the figures and the main manuscript.
(XLSX)

## Abbreviations

EEG: electroencephalography
HD: high density
MI: motion index
MR: motion ratio
RM-ANOVA repeated measures analysis of variance
SAM: stroboscopic alternative motion
SEM: standard error of the mean
STD: standard deviation
RS: resting state
tACS: transcranial alternating current stimulation
