# Supplementary figures and images for: Selective Modulation of Interhemispheric Functional Connectivity by HD-tACS Shapes Perception
Source: PLoS Biol. 2014 Dec 30;12(12):e1002031. doi: 10.1371/journal.pbio.1002031 (PMC4280108; doi:10.1371/journal.pbio.1002031)

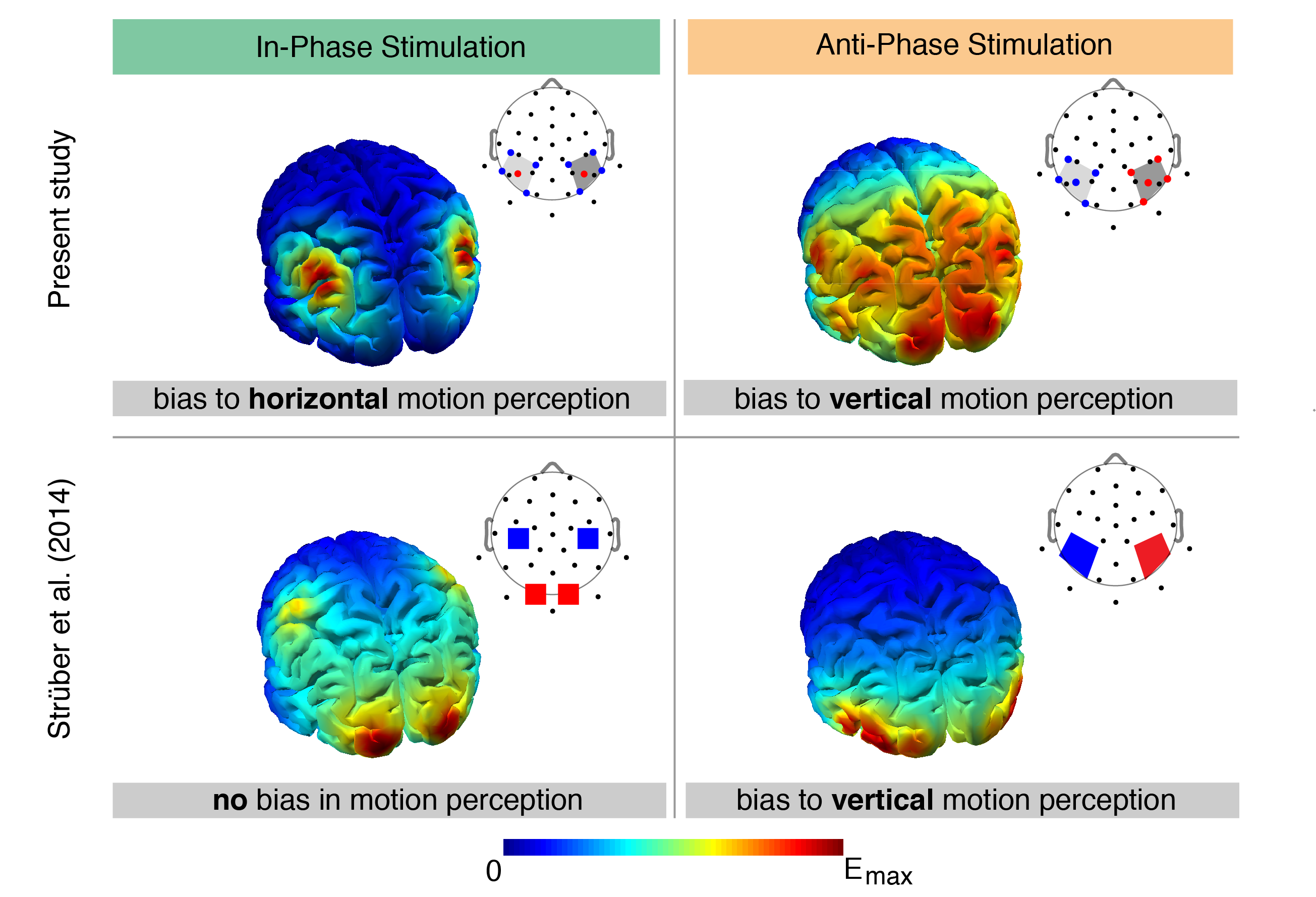

Supplement: Figure S1 — Related to results: electric field simulation. Electric field simulation for the present study (upper row) and the study by Strüber and colleagues (lower row [18]). Simulations on the left depict in-phase stimulation; simulations on the right depict anti-phase stimulation. Stimulation electrode positions are highlighted on the small panel on a 2D topographic map (same color conventions as in Figure 1C). Electrode placement in the study by Strüber and colleagues was according to the international 10–10 system: In-phase stimulation was delivered over C3, C4, and slightly posterior to O1 and O2, while anti-phase stimulation was delivered over P7, PO7, P8, and PO8. In the present study, the stimulation polarity for the anti-phase session was adjusted to closely match the montage as introduced by Strüber and colleagues. The electric field simulations of the present study indicate that both montages targeted the extrastriate visual cortex and induced subsequent behavioral modulations, with the in-phase setup providing a more focal stimulation. In contrast, the anti-phase montage by Strüber and colleagues also targeted the extrastriate cortex and induced subsequent behavioral alterations, while their in-phase montage targeted mainly the occipital pole and induced no subsequent behavioral modulation. Note that only the relative electric field spread is depicted. (TIF) [file pbio.1002031.s001.tif]

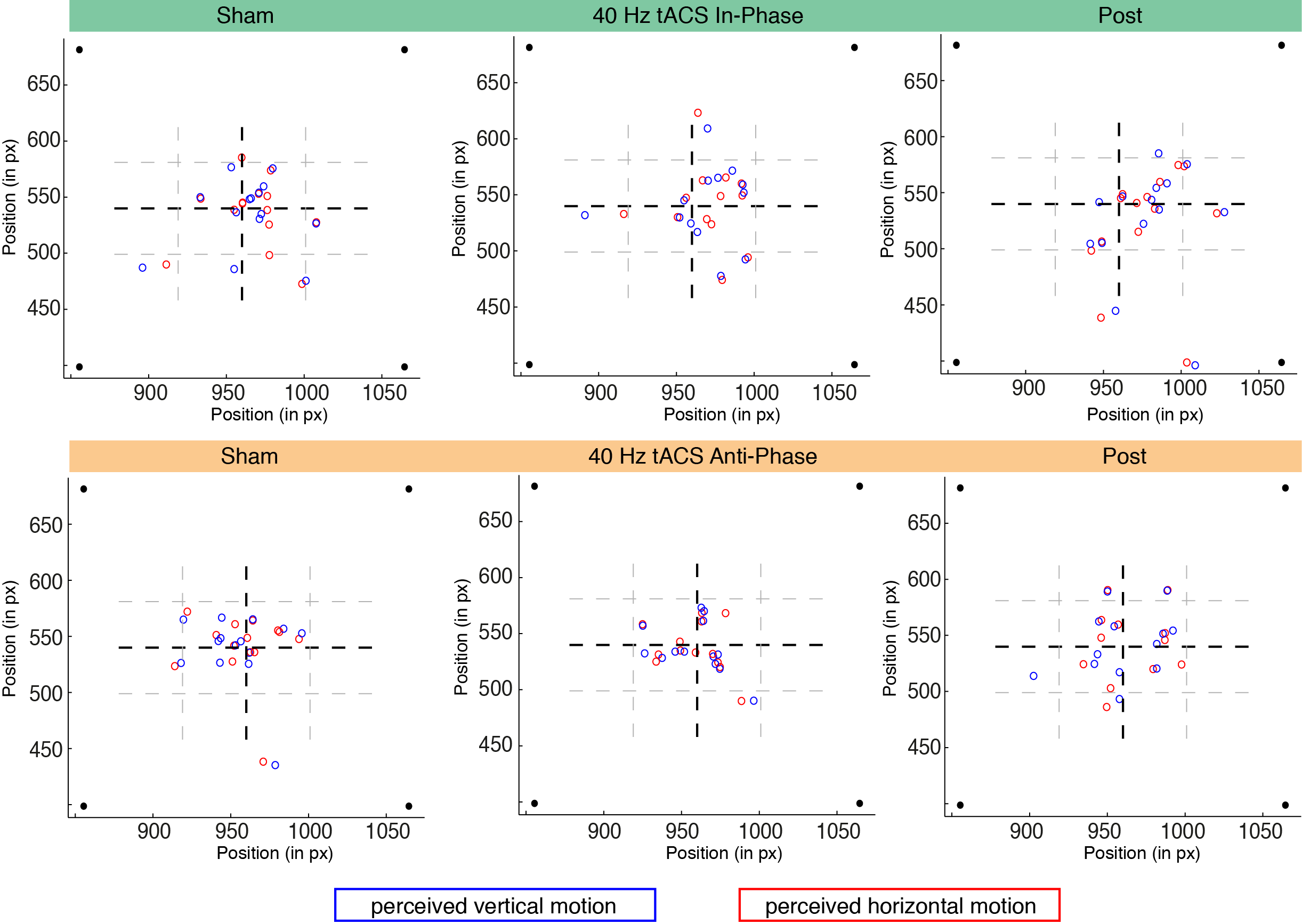

Supplement: Figure S2 — Related to control analysis: eye data on fixation. Mean deviation from fixation cross (positioned centrally at 970×540 pixels) for sham, stimulation, and post conditions during the in-phase (upper) and anti-phase (lower) session. Black dots in corners depict possible positions of the SAM. Grey line spacing is 1° visual angle. Red and blue dots depict each subject's mean position during perceived vertical (blue) and horizontal (red) motion. (TIF) [file pbio.1002031.s002.tif]

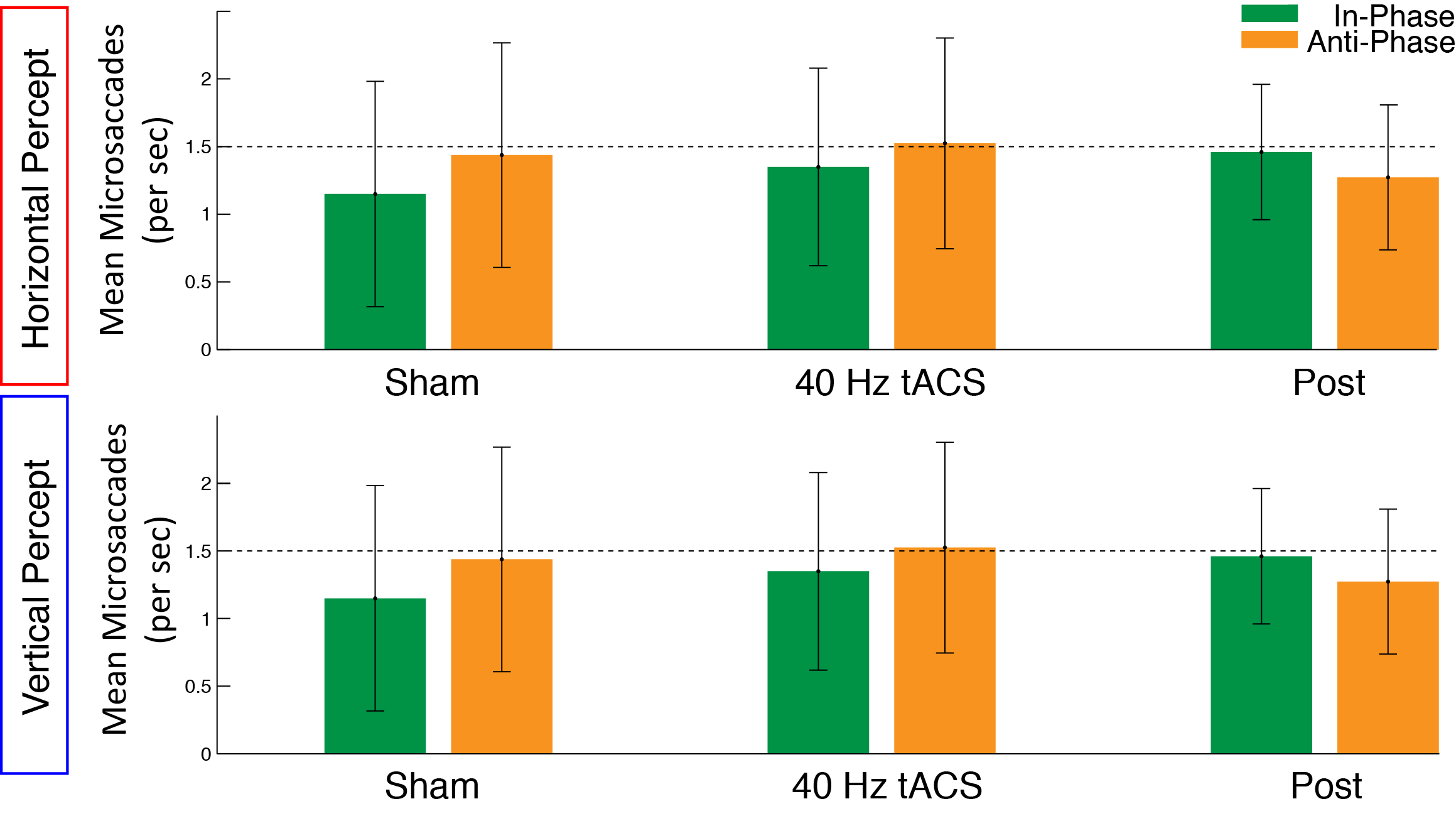

Supplement: Figure S3 — Related to control analysis: eye data on microsaccades. Upper panel: Mean microsaccade rate per second (± STD) for all sessions and conditions during horizontal motion perception. Lower panel: Mean microsaccade rate per second (± STD) for all sessions and conditions during vertical motion perception. Dashed grey lines depict 1.5 microsaccades per seconds. (TIF) [file pbio.1002031.s003.tif]
