# Supplementary material for: Selective Modulation of Interhemispheric Functional Connectivity by HD-tACS Shapes Perception
Source: PLoS Biol. 2014 Dec 30;12(12):e1002031. doi: 10.1371/journal.pbio.1002031 (PMC4280108; doi:10.1371/journal.pbio.1002031)
Supplement: Table S1 — Related to Figure 3: planned contrast analysis. Supplemental behavioral modulation results: Full statistical comparison in a planned contrast analysis according to our hypothesis. In addition, partial eta squared indicates the effects size for all factors. Contrast L1: In- versus anti-phase session; Contrast L2: Sham versus stimulation; Contrast L3: Sham versus post. (DOCX) [file pbio.1002031.s004.docx]

**Table S1 Related to Figure 3: Planned Contrast Analysis**

|  |  |  | df | F-value | p-value | Partial Eta squared |
| --- | --- | --- | --- | --- | --- | --- |
| *Session* |  |  | 1,13 | 0.24 | = 0.63 | 0.018 |
| *Condition* |  |  | 1.3,17.1 | 0.02 | = 0.93 | 0.002 |
| *Session * Conditions* |  |  | 2,26 | 3.36 | **= 0.05** | 0.206 |
|  | **Planned contrasts** | |  |  |  |  |
| *Session* | L1 |  | 1,13 | 0.24 | = 0.63 | 0.018 |
| *Condition* |  | L2  L3 | 1,13  1,13 | 0.003  0.06 | = 0.96  = 0.82 | 0.000  0.004 |
| *Session * Condition* | L1 | L2  L3 | 1,13  1,13 | 5.17  2.70 | **< 0.05**  = 0.12 | 0.285  0.172 |
