# Supplementary material for: Selective Modulation of Interhemispheric Functional Connectivity by HD-tACS Shapes Perception
Source: PLoS Biol. 2014 Dec 30;12(12):e1002031. doi: 10.1371/journal.pbio.1002031 (PMC4280108; doi:10.1371/journal.pbio.1002031)
Supplement: Table S2 — Related to Discussion: comparison of the present study to Strüber and colleagues [1],[18] . Strüber and colleagues [1],[18] were the first to investigate the impact of tACS on ambiguous motion perception. Their results indicated that only anti-phasic stimulation at 40 Hz influenced the conscious experience of apparent motion perception. The authors obtained no significant results for in-phase stimulation at 40 Hz, nor did they find any effects for 6 Hz or sham stimulation. In particular, they demonstrated that anti-phasic 40 Hz tACS increased the amount of perceived vertical motion in the SAM. Here, we replicated their key behavioral finding and adjusted several experimental parameters to demonstrate that interhemispheric coherence could, in fact, be modulated in a desired and predictable fashion. Main differences are outlined in the table above. The most important differences are highlighted in bold type. (DOCX) [file pbio.1002031.s005.docx]

**Table S2 Related to Discussion: Comparison of the Present Study to Strüber et al. (2014)**

|  | Present study | Strüber et al. (2014) |
| --- | --- | --- |
| *Study design* | **Within-group** design, sham-controlled. Group recruited from the University Medical Center in Hamburg, Germany. | **Between-group** design, sham controlled. Group recruited from the University of Oldenburg, Germany. |
| *Procedure* | Volunteers were familiarized with ambiguous (14min) and non-ambiguous (7min) versions of the SAM to reliably track their percept. SAM presentation was organized in trials (1min each) and intermitted by short breaks (few seconds). | Naïve volunteers observed ambiguous and non-ambiguous motion in a training run (each 5min). SAM presentation was continuous. |
| *Stimulus design* | **Stimulus dimensions were 5.1° x 6.9°** to avoid foveal (central 3°) visual stimulation. Vertical distance was larger than horizontal to compensate for the vertical bias of equidistant SAM displays. | An equidistant SAM display (5cm x 5cm) was observed in 130cm distance (equals **2.2° x 2.2°**). Thus, visual stimulation was mainly foveal (within central 3°) and not in the visual periphery. Despite the debate about a bilateral representation of the fovea it seems plausible to assume that interhemispheric integration is more important for stimuli that are further separated in the visual field. |
| *Stimulus presentation* | The SAM was displayed on a computer screen at 120 Hz. Displays changed at a rate of **4 Hz**. | Four light emitting diodes were driven by a NIDAQ device at **2 Hz**. |
| *tACS settings* | All subjects were stimulated with **1 mA**. | **Individual** skin and phosphene **thresholds** were used:  40 Hz Anti-phase: 1.02 ± 0.62 mA  40 Hz In-Phase: 1.23 ± 0.35 mA  6 Hz Anti-Phase: 0.50 ± 0.19 mA  6 Hz In-Phase: 1.40 ± 0.39 mA |
| *Electrode montage* | **Ten** **Ag/AgCl** **electrodes** were used for stimulation.  Anti-Phase: Stimulation electrodes were positioned to match the Strüber study to reproduce and validate previously observed effects.  In-Phase: 4x1 electrode layouts were used to specifically target the extra-striate visual cortex (resulting in a more lateral stimulation). | **Standard tACS electrodes (5x7cm)** were used for the anti-phase experiment. In-Phase stimulation utilized four 3.9x3.9cm electrodes over C3/C4/O1/O2 positions, thus, resulting in a more central electrical stimulation |
| *EEG recordings* | EEG recordings were carried out prior and after stimulation (in total six resting states). A second sham (post) measurement was performed to assess for outlasting effects.  Importantly, **EEG was also recorded and evaluated during tACS.** | **EEG recordings were only carried out before and after tACS.** Outlasting effects (in a second sham) were only assessed during the In-Phase session. |
| *EEG analysis* | EEG recordings during SAM highlighted sources of gamma activity in both parieto-occipital cortices. Spectral estimates were obtained by means of a multi-taper method as implemented in Fieldtrip (Hanning window for 1-35 Hz, multitapers with spectral smoothing for 36-100Hz). Gamma power sources did not differ in their configuration during stimulation. Functional coupling was assessed by means of Magnitude-Squared Coherence and Phase-Locking Values to account for signals that have 0° phase lag across hemispheres. | Spectral power and Magnitude-Squared Coherence (mscohere function, Hamming-window, in MatLab) were assessed in pre- and post-measurements only. |
| *Eyeposition* | Eyetracking was performed to assess influences of fixation and microsaccades on EEG signals. | No eyetracking was performed. EOGs were recorded. |
