# Supplementary material for: Selective Modulation of Interhemispheric Functional Connectivity by HD-tACS Shapes Perception
Source: PLoS Biol. 2014 Dec 30;12(12):e1002031. doi: 10.1371/journal.pbio.1002031 (PMC4280108; doi:10.1371/journal.pbio.1002031)
Supplement: Text S1 — Related to Figure 1C: electric field simulation. Supporting information for the modeling of the electric field. Results of the modeling can be found in Figure S1. (DOCX) [file pbio.1002031.s006.docx]

**Text S1 Related to Figure 1C: Electric Field Simulation**

The electric field distribution during electrical brain stimulation, where currents are inserted and extracted at specified scalp electrode locations, is equivalent to the forward calculation for cortical sources of scalp EEG activity, and can therefore be expressed in terms of the electric lead field. The lead field $\vec{L}\left( \vec{r} \right)$ is a vector field defined for a specific electrode and reference such that for a current dipole with moment $\vec{D}(\vec{r})$ the electric potential between electrode and reference is given by

If the electric potential is known for all sources, the k^th^ component of the lead field at a specific location is defined by the electric potential induced by a unit dipole in the k^th^ direction at that position.

The electric lead field is identical to the electric field induced by a unit current inserted at the reference and extracted at the electrode. From this basic relation, the electric field for an arbitrary current inserted and extracted (equals an insertion with a negative sign) can be calculated at a set of electrodes by superposition. With α_i_ being the current inserted at the i^th^ electrode and the lead field $\vec{L}_{i}\left( \vec{r} \right)$ for the i^th^ electrode, the electric field inside the brain was calculated as

Here, the electric forward problem was solved using an analytic expansion of the electric lead field [1] for a 3-shell volume conductor containing scalp, skull and brain, taken from the MNI standard head. The conductivity ratio between skull and scalp, and also between skull and brain, was chosen to be 1:50 selected as a compromise between the standard value 1:80 and more recent findings suggesting much smaller ratios [2,3].

1. Nolte G, Dassios G (2005) Analytic expansion of the EEG lead field for realistic volume conductors. Phys Med Biol 50: 3807–3823. doi:10.1088/0031-9155/50/16/010.

2. Miranda PC, Lomarev M, Hallett M (2006) Modeling the current distribution during transcranial direct current stimulation. Clin Neurophysiol 117: 1623–1629. doi:10.1016/j.clinph.2006.04.009.

3. Wagner S, Rampersad SM, Aydin U, Vorwerk J, Oostendorp TF, et al. (2014) Investigation of tDCS volume conduction effects in a highly realistic head model. J Neural Eng 11: 016002. doi:10.1088/1741-2560/11/1/016002.
